# Ni^2+^-Assisted Hydrolysis May Affect the Human Proteome; Filaggrin Degradation *Ex Vivo* as an Example of Possible Consequences

**DOI:** 10.3389/fmolb.2022.828674

**Published:** 2022-03-10

**Authors:** Ewa Izabela Podobas, Danuta Gutowska-Owsiak, Sébastien Moretti, Jarosław Poznański, Mariusz Kulińczak, Marcin Grynberg, Aleksandra Gruca, Arkadiusz Bonna, Dawid Płonka, Tomasz Frączyk, Graham Ogg, Wojciech Bal

**Affiliations:** ^1^ Institute of Biochemistry and Biophysics, Polish Academy of Sciences, Warsaw, Poland; ^2^ Medical Research Council Human Immunology Unit, National Institute for Health Research Oxford Biomedical Research Centre, Medical Research Council Weatherall Institute of Molecular Medicine, University of Oxford, Oxford, United Kingdom; ^3^ Institute of Genetics and Biotechnology, University of Warsaw, Warsaw, Poland; ^4^ University of Gdansk, Intercollegiate Faculty of Biotechnology of University of Gdansk and Medical University of Gdansk, Gdansk, Poland; ^5^ SIB Swiss Institute of Bioinformatics, Vital-IT Team, Lausanne, Switzerland; ^6^ The Maria Sklodowska-Curie National Research Institute of Oncology, Warsaw, Poland; ^7^ Institute of Informatics, Faculty of Automatic Control, Electronics and Computer Science, Silesian University of Technology, Gliwice, Poland; ^8^ Department of Biochemistry, University of Cambridge, Cambridge, United Kingdom

**Keywords:** filaggrin, human proteome, protein degradation, Ni^2+^-assisted hydrolysis, nickel toxicity, nickel allergy

## Abstract

Deficiency in a principal epidermal barrier protein, filaggrin (FLG), is associated with multiple allergic manifestations, including atopic dermatitis and contact allergy to nickel. Toxicity caused by dermal and respiratory exposures of the general population to nickel-containing objects and particles is a deleterious side effect of modern technologies. Its molecular mechanism may include the peptide bond hydrolysis in X_1_-S/T-c/p-H-c-X_2_ motifs by released Ni^2+^ ions. The goal of the study was to analyse the distribution of such cleavable motifs in the human proteome and examine FLG vulnerability of nickel hydrolysis. We performed a general bioinformatic study followed by biochemical and biological analysis of a single case, the FLG protein. FLG model peptides, the recombinant monomer domain human keratinocytes *in vitro* and human epidermis *ex vivo* were used. We also investigated if the products of filaggrin Ni^2+^-hydrolysis affect the activation profile of Langerhans cells. We found X_1_-S/T-c/p-H-c-X_2_ motifs in 40% of human proteins, with the highest abundance in those involved in the epidermal barrier function, including FLG. We confirmed the hydrolytic vulnerability and pH-dependent Ni^2+^-assisted cleavage of FLG-derived peptides and FLG monomer, using *in vitro* cell culture and *ex-vivo* epidermal sheets; the hydrolysis contributed to the pronounced reduction in FLG in all of the models studied. We also postulated that Ni-hydrolysis might dysregulate important immune responses. Ni^2+^-assisted cleavage of barrier proteins, including FLG, may contribute to clinical disease associated with nickel exposure.

## Introduction

**FIGURE 1:**
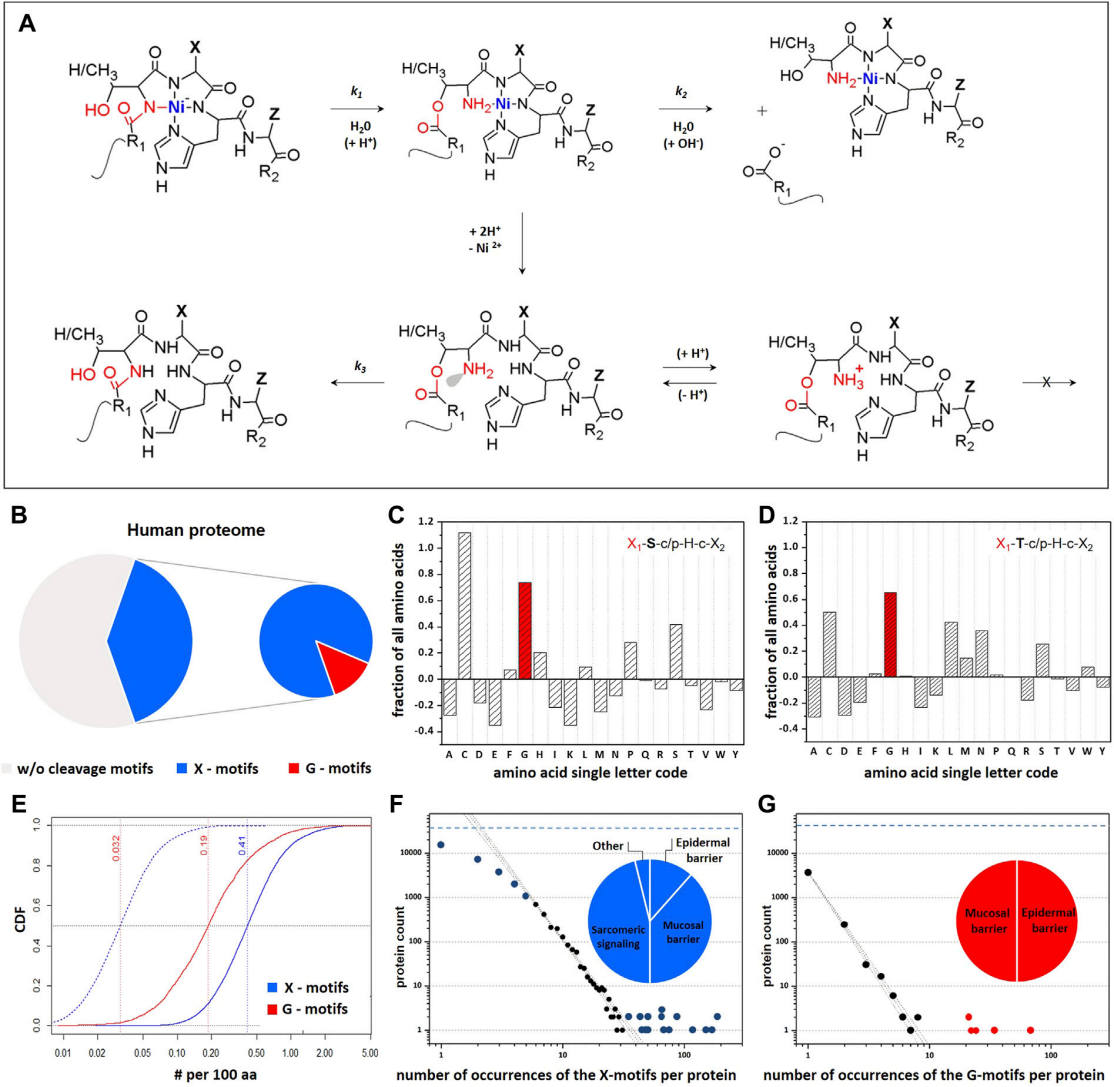
Frequency of Ni-hydrolysis-susceptible X_1_-ST-cp-H-c-X_2_ motifs (Kopera et al., 2010; Krezel et al., 2010; Podobas et al., 2014) within the human proteome. **(A)** Ni-hydrolysis is enabled through formation of a square-planar 4N Ni^2+^ complex involving the imidazole nitrogen of the participating histine and three preceding amide nitrogens. The N–O acyl shift from the carbonyl group of S/T peptide bond to the hydroxyl group, follows an apparent first order kinetic regime **(k**_**1**_ **)**. The resulting ester hydrolyzes spontaneously into two peptides, also according to the apparent first order kinetics **(k**_**2**_ **)**. Protonation of metal ion binding to the amine group prevents the O–N acyl shift by engaging the nitrogen’s lone electron pair (grey). The Ni^2+^ ion facilitates the acyl shift and prevents its reversal, by stabilization of the reaction products. **(B)** Schematic percentage representation of the Ni-assisted hydrolysis-susceptible motifs within human proteome. **(C,D)** Propensities of each of the 20 canonical amino acids on X_1_-position within S and T containing motifs. Frequencies of individual amino acids on X_1_-positon were corrected for their occurrence in the whole proteome. Positive values identify amino acids that are more likely to occur in this position than in the total proteome. **(E)** Cumulative distribution function (CDFs) of G occurrences on the X_1_ position within hydrolytic motifs (red). Distribution is normalized per 100 residues in protein sequences. Chopped blue line follows the CDF expected for G-motif, which was obtained from the CDF observed for X-motif (blue line) after correction for 7.07% of glycine content. Each individual protein is contributing to the CDF curves as a single step of the same height. Dotted vertical lines denote the median of the distribution. **(F,G)** Quantitative analysis of the number of hydrolytic motifs in each protein. Thick lines follow the general trend expected according to the Poisson distribution used for statistical analysis, while dashed lines indicate the associated 95% confidence bands. Blue and red symbols mark data substantially deviated from Poisson distributions. The total number of proteins with X-motifs in the database (31099, dashed horizontal line) stands the upper limit for the number of proteins with hydrolytic motifs, thus explaining the deviation observed for proteins with small number of cleavage motifs occurrences.

Prevalence of nickel alloys in the industry and daily use items is inadvertently associated with the occupational and environmental exposure to airborne particles containing nickel oxides and salts, and to Ni^2+^ ions present in water and food and released from nickel alloys (by dermal contact) (World Health Organization, 2000; Kasprzak et al., 2003; Nieminen et al., 2007; Zambelli et al., 2016; Ahlström et al., 2019). While medicinal aspects of the resulting nickel toxicity have been thoroughly described, the underlying molecular mechanisms remain the subject of research (Ahlström et al., 2019; Genchi et al., 2020). The Ni^2+^-assisted peptide bond hydrolysis (Ni-hydrolysis) is one such reaction, occurring selectively before S/T in proteins bearing X_1_-S/T-c/p-H-c-X_2_ motifs (Ni-hydrolytic motifs, excluding P at the third and reduced C at the first, third and fifth residues within the motif) exposed to Ni^2+^ ions in solution (Kopera et al., 2010; Krezel et al., 2010; Podobas et al., 2014). It proceeds via the N-O acyl shift in the X_1_-S/T moiety, followed by ester hydrolysis (Figure 1A). The reaction rate depends on pH, temperature and the bulkiness of the first, third and fifth residues, being significantly faster for X_1_ = G (fast motifs) (Ariani et al., 2013). The effectiveness this process was proven for Cu^2+^ (Bal et al., 2000) and Pd^2+^ ions (Wezynfeld et al., 2016), but Ni-hydrolysis was investigated to the largest extent, due to its higher efficiency. On the other hand, Co^2+^ and Zn^2+^ ions were proven to be non-reactive in this respect (Bal et al., 2000).

Filaggrin (FLG) plays key roles in maintaining skin homeostasis, epidermal structure and the barrier function (Candi et al., 2005; Sandilands et al., 2009; Brown and McLean, 2012). It is expressed as a large >400 kDa precursor (profilaggrin; proFLG), forming the bulk of keratohyalin granules (KHGs). ProFLG consists of 10–12 nearly identical FLG repeats, subsequently released from KHGs into the cytoplasm during post-translational processing (Matoltsy and Matoltsy, 1970). These monomers are essential in aggregating keratin filaments in *stratum corneum* while the proFLG N-terminal domain plays an important role during epidermal terminal differentiation process. Further FLG proteolysis proceeds down to amino acids, which contribute to the epidermal hydration, acidic *stratum corneum* pH maintenance, and protection against UV radiation (Gibbs et al., 2008; Kezic et al., 2008; Fluhr et al., 2010). FLG release from the KHGs (Gutowska-Owsiak et al., 2018) and processing is precisely controlled spatially and temporally, and the FLG deficiency results in abnormal epidermal architecture and barrier insufficiency, promoting skin inflammation and allergic sensitisation by allergens penetrating the defective epidermis (Gruber et al., 2011). Finally, FLG loss-of-function mutations might also increase dermal absorption of chemicals (Rietz Liljedahl et al., 2021) and increase the risk for allergic sensitization against nickel (Novak et al., 2008; Thyssen et al., 2010).

The key role of FLG in the skin barrier formation and its enrichment in Ni-hydrolytic motifs inspired us to study its interaction with Ni^2+^ ions in cell-free and biological systems.

Here we present the hydrolytic cleavage pattern search tool which allowed us to analyse the distribution of such cleavable motifs in the human proteome and catalogued them according to their physiological function. Fast G-motifs are generally abundant, but particularly highly enriched in proteins supporting the epidermal barrier of the human body, e.g. FLG, expressed predominantly in keratinocytes.

## Materials and Methods

### Database Creation

For our *in silico* research the UniProt database—release 2017_05—was used. We decided to leave all protein isomers as a full representation of functional proteome. But we keep only a non-redundant protein set to avoid duplicates after clustering with the cd-hit tool version 4.6.8 and these parameters: “-c 0.98 -d 0 -aS 0.98 -p 1 -g 1”. Two patterns—following the PROSITE Pattern syntax (https://prosite.expasy.org/prosuser.html)—were searched with the/Pattern Search/ tool version 1 from MyHits (https://www.ncbi.nlm.nih.gov/pubmed/17545200): “x-[ST]-{CP}-H-{C}-x” or “G-[ST]-{CP}-H-{C}-x”. Perl 5.18.2 (script get_motif_stat.pl) was used to parse results. Additionally results containing proteins with those words in their description were discarded: “(Fragment)” or “Truncated”. Here a correction has been made. In the case of overlapping motives, we counted them as one. The following research tasks were considered: estimation of the number of motifs per protein, estimation of the number of motifs normalized by the length of proteins, estimation of the number of motifs by type (specific amino acids at specific positions). On the base of such prepared data, further statistical analyses were performed.

### Amino Acid Enrichment

Experimental amino acid occurrences on position X_1_ of X_1_-S/T-c/p-H-c-X_2_ motif were corrected for the abundance of each amino acid in the whole proteome. Positive value indicates that a particular residue type is found on X-position more frequently than in the whole proteome.

### Cumulative Distribution Function (CDF)

Cumulative distribution functions were obtained according to the standard approach (Montgomery and Runger, 2007) and visualized using Origin software (version 9.7, www.originlab.com).

### Quantitative Analysis of Hydrolytic Motifs Frequency Within the Human Proteome

The number of X_1_-S/T-c/p-H-c-X_2_ motifs was determined for each individual protein. The distribution of these numbers was then analysed assuming Poisson distribution, which assumes no correlations between particular motifs. The analysis was performed using Origin software (version 9.7, www.originlab.com).

### Single-Term GO Functional Enrichment Analysis

Gene Ontology functional enrichment analysis was performed using topGO R package with custom GO mapping files based on Gene Ontology human annotations files from April 2018. Only Gene Ontology terms from Biological Process were used for annotation. Initial datasets G-S/T-c/p-H-c-X_2_ and X_1_-S/T-c/p-H-c-X_2_ included 4111 and 31099 proteins denoted with UniProt accession numbers. As GO annotations are provided for UniProtKB identifier, the first step involved translation UniProt accession numbers to Uniprot KB identifiers and filtering out isoforms. After the filtering step, the datasets G-S/T-c/p-H-c-X_2_ and X_1_-S/T-c/p-H-c-X_2_ 2938 and 23120 protein identifiers respectively. To assess the statistical significance of GO terms enrichment in both datasets, Fisher Exact test was used with Benjamin-Hochberg adjustment for multiple testing corrections.

### Analysis of Hydrolytic Motifs Within Filaggrins From Various Species

In order to create the list of proteins from diverse organisms we used the ScanProSite Tool which allows to scan a protein database against a motif. We used the X_1_-S/T-c/p-H-c-X_2_ motif for this purpose. We then normalized the number of cleavage sites dividing it by the total sequence length (# of motifs * 100/seq length). Dataset has been used to create a circular phylogenetic tree in NCBI CommonTree (https://www.ncbi.nlm.nih.gov/Taxonomy/CommonTree/wwwcmt.cgi) and visualized in iTOL https://itol.embl.de/(Letunic and Bork, 2021).

### *In Silico* Analysis of proFLG Sequence and Selection of Peptides

In silico analysis of proFLG sequence was performed on the base available in NCBI database human proFLG amino acid sequence (Ref. NP_002007.1) and information about FLG repeats (Sandilands et al., 2009). The FLG domains separation was proposed and compared using WebLogo 3 application (Schneider and Stephens, 1990; Crooks et al., 2004).

### Peptide Synthesis

All peptides were synthesized in the solid phase according to the Fmoc protocol (Chan and White, 2000) using the Prelude automatic synthesizer (Protein Technologies). The syntheses were accomplished using N-α-9-Fluorenylmethyloxycarbonyl (F-moc) amino acids (Novabiochem) on a TentaGel^®^ S RAM resin (Rapp Polymere), using O-(Benzotriazol-1-yl)-N,N,N′,N′-tetramethyluronium hexafluorophosphate (HBTU, Sigma-Aldrich) as a coupling reagent, in the presence of N,N-diisopropylethylamine (DIEA, Sigma-Aldrich). The acetylation of the N-terminus was carried out in 10% acetic anhydride in DCM. The cleavage was done manually by the cleavage mixture composed of 95% trifluoroacetic acid (TFA, Sigma-Aldrich), 2.5% triisopropylsilane (TIS, Sigma-Aldrich) and 2.5% water. Peptides were isolated from cleavage mixtures by the addition of ice-cold diethyl ether and centrifugation. Following precipitation, peptides were dissolved in water and lyophilized. Peptides were purified by HPLC (Waters) using an analytical C18 column (ACE 250 × 4.6 mm) monitored at 220 and 280 nm. The eluting solvent A was 0.1% (v/v) TFA in water, and solvent B was 0.1% (v/v) TFA in 90% (v/v) HPLC grade acetonitrile (Rathburn Chemicals). The correctness of molecular masses and purities of the peptides was confirmed using Premier Q-Tof ESI-MS spectrometer (Waters). After this step, peptide solutions were frozen in liquid nitrogen and lyophilized.

### Ni^2+^-Assisted FLG Peptides Hydrolysis

The hydrolysis experiments were performed in a 20 mM HEPES buffer (Sigma-Aldrich), using 0.5 mM peptide and 2 mM Ni(NO_3_)_2_ (Sigma-Aldrich). The samples were incubated in pH 8.2, at 50°C and pH 7.4, at 37°C. The aliquots were periodically collected from the samples and acidified by addition 2% (v/v) TFA. Control samples, containing peptide and buffer, but without Ni^2+^, were gathered at the same time points. For analysis, reaction mixtures were diluted by water 4 to 1 and injected into the HPLC system (Waters), equipped with an analytical C18 column. The eluting solvent A was 0.1% (v/v) TFA in water, and solvent B was 0.1% (v/v) TFA in 90% (v/v) acetonitrile. The chromatograms were obtained at 220 and 280 nm. After separation, the products of hydrolysis were identified using electrospray ionization mass spectrometry (ESI-MS). The relative amounts of these fractions in each chromatogram were calculated by peak integration using data analysis software Origin 8.1 or Origin Pro 2017 (OriginLab Corporation).

### Kinetic Analysis

To calculate the rate constants of the acyl shift step (*k*_1_) and the ester hydrolysis step (*k*_2_) of the hydrolysis reaction the set of three equations (Kinet A, Kinet B, and Kinet C) was used, similarly to previous studies (Kopera et al., 2010; Ariani et al., 2013; Protas et al., 2013).

$$\text{Kinet}\;\text{A}\;y=A_{0}\times \text{exp}\left(-k_{1}\times x\right)$$

$$\text{Kinet}\;\text{B}\;y=\left(\frac{k_{1}\times A_{0}}{k_{2}-k_{1}}\right)\times \left(\exp\left(-k_{1}\times x\right)-\exp\left(-k_{2}\times x\right)\right)$$

$$\text{Kinet}\;\text{C}y=A_{0}\times \left(1+\left(\frac{1}{k_{1}-k_{2}}\right)\right)\times \left(k_{2}\exp\left(-k_{1}\times x\right)-\exp\left(k_{2}\times x\right)\right)$$

In these equations *y* is a molar fraction of a given species, *x* is the time axis, and A_0_ denotes the initial concentration of the substrate.

### UV−Visible and Circular Dichroism Spectroscopies

The UV-visible spectra were recorded in the range of 850–330 nm, on a LAMBDA 950 UV/vis/NIR spectrophotometer (PerkinElmer). The path length was 1 cm. Complexometric titrations were performed for the samples containing 0.95 mM peptide and 0.9 mM Ni(NO_3_)_2_ dissolved in H_2_O. The pH of the solution was adjusted manually in the range of 3–11.5 by titrating with small amounts of concentrated NaOH. Circular dichroism (CD) spectra of Ni^2+^ complexes with peptides were recorded in the range of 270–800 nm, on a Jasco J-815 spectropolarimeter, using the same samples as for UV-vis experiments. The p*K*_a_ values for the complex formation were obtained by fitting the absorption value at the band maximum to the Hill equation (Acerenza and Mizraji, 1997).

### Molecular Modeling of Ni^2+^ Complexes

All molecular mechanics simulations were performed using YASARA2 force-field (Krieger et al., 2004) extended for the Ni^2+^ coordination by adding the *ab initio* derived topology and charge distributions. The N-Ni distances were constrained as a pseudo-bond of the appropriate length, and the geometry was forced according to the expected square planar coordination of Ni^2+^ by additional constrains for N-Ni-N angles (90°) and N-Ni-N-N pseudo-dihedrals (180°). Additional pseudo-dihedral constrains were introduced to mimic sp^2^ hybridisation of the Nitrogen (C-N-Cα-N = 180°). Model peptides were built in extended conformation and the structure of their complexes with Ni was initially optimised using implemented in Yasara algorithm that combines simulated annealing and energy minimisation. The further ten cycles of high temperature molecular dynamics (250 ps at 1,000 K) followed by stepwise cooling and final energy minimization were done to assess conformational flexibility of a given Ni-peptide complex. Molecular graphics were created with YASARA (www.yasara.org) and POVRay (www.povray.org).

### FLG Recombinant Protein: Plasmid Construction

Plasmids were constructed using a sequence- and ligation-independent cloning (SLIC) method (Li and Elledge, 2007). Nucleotide sequence encoding 10th FLG repeat domain were encloned in pET28 vector using BamHI and XhoI restrictions sites. The FLG 10th construct contained a C-terminal His6-tag. As a control, a construct for maize protein kinase CK2α was obtained in similar conditions. The CK2α protein has a molecular weight similar to FLG and contains no X_1_-S/T-c/p-H-c-X_2_ motifs. The maize protein kinase CK2α construct contained a C-terminal His6-tag. The constructs were verified by sequencing.

### Protein Production and Purification

The constructs were transformed into *E. coli* BL21-CodonPlus-RIL and propagated overnight in LB liquid media containing kanamycin and chloramphenicol at 37°C. The bacterial cultures were diluted 1:100 in LB liquid media supplemented by antibiotics and incubated at 37°C until the culture has reached the mid-log phase of growth. Protein expression was induced by IPTG (1 mM) for 2 h at 37°C. The cells were harvested by centrifugation (10 min, 5,000 × *g*, 4°C). The pellets were mixed with lysis buffer (10 mM Tris pH 8, 150 mM NaCl, 10 mM imidazole) supplemented with protease inhibitors cocktail and lysed by sonication. The cell lysate was clarified by centrifugation (60 min, 24,000 × *g*, 4°C) and used for affinity purification on a HisPur™ Cobalt Resin (Thermo Fisher). Pure protein was eluted by an elution buffer (10 mM Tris pH 8, 150 mM NaCl, 300 mM imidazole). Samples were dialysed (10 mM Tris pH 8.5, 150 mM NaCl, MWCO: 12–14000 Da) and analyzed on SDS-PAGE. Bands of interest were cut out and identified by MALDI-TOF MS after trypsin digestion.

### Ni^2+^-Assisted FLG Monomer Hydrolysis

FLG protein domains (30 µM) were incubated in 10 mM TRIS/150 mM NaCl buffer with or without nickel ions [1 mM Ni(NO_3_)_2_] under optimal (pH 8.2, 50°C) and physiological (pH 7.4, 37°C) conditions. The reactions were stopped by freezing the collected samples in liquid nitrogen. Samples from different time points were separated using the Tricine-SDS page technique and Bio-Rad system. The experiment was repeated for CK2α control protein. Gels after electrophoresis were scanned (E-gel imager Camera, Life Technologies) and the scans used for densitometric analyses (ImageJ program).

### Cell Proliferation Assay

In order to find out the toxic concentration of Ni(NO_3_)_2_ for keratinocytes, the cell proliferation assay (MTT) was performed. Cells were cultured in a 96-well plate and after 24 h of exposure to a gradient of Ni(NO_3_)_2_ concentrations (10^−2^ to 10^−7^ M final concentration) the test was performed according to manufacturer’s protocol (CellTiter 96^®^ Non-Radioactive Cell Proliferation Assay, Promega). The assay determined the half maximal inhibitory concentration value IC_50_ as approximately 1 mM Ni(NO_3_)_2_.

### Normal Human Epidermal Keratinocyte Culture

Normal human epidermal keratinocytes (NHEKs) (purchased from Lonza) were cultured in monolayers in a dedicated medium (Lonza, KBM-2) at the Ca^2+^ level of 0.06 mM. To stimulate differentiation and FLG expression, a calcium switch was conducted over a period of 24 h by replacing the culture media with fresh media adjusted to a 1.5 mM final calcium concentration. A Ni(NO_3_)_2_ (Sigma) solution was added to achieve various final concentrations (10 μM, 100 μM and 1 mM). Doses were chosen based on MTT test results (Supplementary Figure S1). After 24 h of incubation, the cells were fixed, permeabilized and immunostained with anti-FLG antibodies (Anti-FLG goat G20 (Santa Cruz), and secondary anti-goat Alexa-488 and anti-rabbit Alexa-568 (Life Technologies) antibodies were used. Staining was carried out in PBS and nuclei were visualized by Hoechst (NucBlue, Life Technologies). The slides were coversliped with Mowiol 4-88 (Sigma). Data acquisition was carried out on the Zeiss 780 inverted confocal microscope. Images from three separate experiments were analysed; KHG diameter and integrated intensity from the signal were measured using Fiji: ImageJ program (Abramoff, 2007). For the statistical analysis the Mann–Whitney *U* test was used.

### Exposure of Epidermal Sheets to Nickel

Skin samples were obtained from healthy donors undergoing surgery under ethical approval from the UK National Research Ethics Service (14.NW.1153). Epidermal sheets were separated from dermal tissues by overnight incubation in dispase (5 U/ml; Sigma Aldrich) and cultured up to 48 h in KGM-2 keratinocyte medium (Lonza) adjusted with CaCl_2_ to a 1.5 mM final calcium concentration. The Ni(NO_3_)_2_ solution was added at the 1 mM final concentration. Experiments were repeated on skin explants from 10 donors. For fluorescent antibody staining epidermal sheets were fixed with 4% formaldehyde (Sigma), followed by 0.1% Triton X-100 (Sigma Aldrich) and incubated in a blocking buffer (5% FCS, 2% BSA in PBS) for 1 h. Anti-FLG goat G20 (Santa Cruz), and the secondary anti-goat Alexa488 (Life Technologies) antibody staining was carried out in PBS for 1 h. The nuclei were visualized by Hoechst (NucBlue, Life Technologies). The sheets were mounted on microscope cover-slides with Mowiol 4-88 (Sigma) for imaging. Data acquisition was carried out on the Zeiss 780 inverted confocal microscope by recording z-stacks of 2D images (at 0.38 µM intervals) and images taken using inverted confocal microscope (Zeiss 780) by recording 2D images in a 3D z-stack.

### Western Blot Analysis

Isolated epidermal sheets were washed in PBS and incubated in an 8M urea buffer (ReadyPrep™ Sequential Extraction Kit, Reagent 2 with reducing reagent; Bio-Rad) and sonicated in a water bath for 30 min. Lysates were spun at 4°C (13,000 rpm, 15 min). Proteins from supernatants were fractionated 7% Tris-Acetate NuPage gels (Life Technologies). Proteins were transferred onto PVDF membranes (iBlot Dry Blot system stacks and iBlot transfer device; LifeTechnologies). Membranes were blocked in a 5% solution of non-fat milk powder in PBS and incubated overnight with desired primary antibodies (anti-FLG goat G-20 (Santa Cruz). Li-Cor infrared secondary antibodies and Li-Cor scanning system (Li-Cor Biosciences) were used for detection.

### RNA Isolation and the Assessment of RNA Integrity

Isolation of total RNA from N-HEK cells was performed using the PureLink^®^ RNA Mini Kit (Ambion) according to the manufacturer’s protocol. The RNA concentration and purity was estimated using NanoDrop 2000 (Thermo). The assessment of RNA quality was carried out on the Agilent 2100 Bioanalyzer System and Eukaryote Total RNA Nano Assay kit (Agilent) was used, according to the manufacturer’s protocol.

### Reverse Transcription

Isolated RNA was used as a template for the reverse transcription. The reaction was performed in a S1000™ Thermal Cycler (Bio-Rad) in 20 µl using the High-Capacity cDNA Reverse Transcription Kit (Applied Biosystem) according to the manufacturer’s protocol. The ingredients contained: 1,000 ng RNA per sample, reaction buffer, random primers, mix of dNTPs, RNase inhibitor (1.0 U/μl) and MultiScribe™ reverse transcriptase (2.5 U/μl). Conditions of RT reaction are presented in the table below.

### Quantitative Real-Time PCR

Gene expression assay was counducted using the StepOne™ Real-Time PCR System and the TaqMan^®^ Gene Expression Assay (Applied Biosystems). Briefly the reaction (holding stage I: 2 min, 50°C; holding stage II: 5 min, 95°C; Cycling stage (40x): 15 s, 95°C and 1 min, 60°C) was performed in 10 µl total volume with 12.5 ng of cDNA (or water as a negative control) addition. TaqMan^®^ Universal Master Mix II with the AmpErase^®^ UNG (uracil-N-glycosylase) (Applied Biosystems) was used. The primers (forward: 5′–GGA​AAA​GGA​ATT​TCG​GCA​AAT–3′, reverse: 5′–TCC​ATG​AAG​ACA​TCA​ACC​ATA​TCT​G–3′) and the TaqMan^®^ MGB probe (5′–FAM CTGAAGAATCCAGATGAC-NFQ-MGB–3′, Applied Biosystems) set for *FLG* gene was designed using Primer Express software (Applied Biosystems) and the specificity was checked using PrimerBLAST tool (NCBI). The specificity of the primers and probe set was confirmed by adequate negative controls. Results were calculated based on a ΔC_T_ method and *TBP1* (TATA-box binding protein 1) gene (commercially available primer and probe set, accession number: Hs00427620, Applied Biosystems) was used as a reference. For statistical analyses the *t*-test was used.

### Langerhans Cell-like Cells

Peripheral blood mononuclear cells (PBMCs) were isolated from blood collected from healthy adult donors under local ethics approval (09/H0606/71). Samples were diluted and centrifuged in a density gradient using a Lymphoprep™ reagent (STEMCELL Technologies Inc.). The CD14^+^ cells were separated with a MACS MicroBead (Miltenyi Biotec) magnetic separation system according to the manufacturer’s protocol. Subsequently, the monocytes were cultured in a 1 × 10^6^/ml density in the RPMI medium supplemented with 10% fetal calf serum, penicillin/streptomycin mix and 2 mM L-glutamine, with addition of cytokines: 250 ng/ml GM-CSF, 100 ng/ml IL-4, 10 ng/ml TGF-β1, all obtained from Pepro-Tech. After 5 days of culture, the cells were exposed to a Ni(NO_3_)_2_ solution (1 mM), peptides (50 µM) or Ni^2+^-peptide complexes with the same concentrations of peptides and of the nickel salt. After 48 h the cells were harvested for the flow cytometry analysis.

### Flow Cytometry

The cells were harvested by decantation into a conical tube and centrifuged (10 min, 1,400 rpm, 4°C). Supernatants were collected and frozen until further analysis. Next, the cells were washed in ice cold 10% FCS in PBS and stained. All staining was carried out on ice and protected from light. Conjugated primary antibodies: anti-human CD86 (APC) and HLA DR (PE), CD80 (FITC) were added in 0.1–10 μg/ml concentration range and incubated for 1 h in the dark at 4°C. The cells were washed three times in PBS and centrifuged (5 min, 1,400 rpm, 4°C), and resuspended in 1 ml of ice cold PBS, containing 10% FCS and 1% sodium azide. The cells were fixed in 1% paraformaldehyde solution and kept in the dark on ice until the analysis. The cytometric analysis was performed on CyAn™ ADP (Beckman Coulter). First, using unstained cells and compensation beads (Anti-Mouse Ig, κ/Negative Control Compensation Particles Set, BD), the compensation procedure was performed. The FCS Express 7 Flow Cytometry Software—RUO, DeNovo Software were used for the final analysis. The results were analysed with the *t*-test.

### Analysis of Cytokine Secretion

Cytokine levels (TNFa, IFNa2, IL1b, IL-6, IL-8, IL-10) in the cultures medium was measured by the Luminex 200™ System (Merck Millipore) and Milliplex HCYTOMAG-60K-07 Human Cytokine MAGNETIC Kit (Merck Millipore). The assay was performed according to the manufacturer’s protocol. The results were analysed with the *t*-test.

## Results

### Ni-Hydrolytic Motifs Are Common in the Human Proteome and Enriched Within Sequences of the Epidermal Barrier Proteins

Our first goal was to characterize and catalogue the distribution of Ni-cleavable motifs within amino acid sequences of human proteins. To this end, the initial UniProt data were cleaned by suitable word filters to eliminate duplicates (partial, truncated or fragmented proteins), while all protein isomers were included to obtain full representation of the functional proteome. This initial data set of 79,077 proteins was searched for the general X_1_-S/T-c/p-H-c-X_2_ motifs and for the particularly interesting G-S/T-c/p-H-c-X_2_ motifs, obtaining 31,099 (Supplementary Table S1) and 4,111 (Supplementary Table S2) records, respectively. We also prepared complementary lists of proteins without X- (Supplementary Table S3) and G- (Supplementary Table S4) motifs. We then determined the absolute number of motifs per protein, their frequency (the count normalized by the length of a given protein, Supplementary Table S1, S2) and the number of motifs by the type (Supplementary Tables S5, S6).

Overall, we determined that as many as 40% of human proteins contain at least one Ni-hydrolytic motif, and 5% of all human proteins contain at least one fast motif (Figure 1B **)**. The analysis of amino acid frequencies in these motifs revealed a significant overrepresentation of X_1_ = G in both S and T motif variants (Figures 1C,D). The cumulative distribution function (CDF) of X_1_ = G illustrates this finding (Figure 1E).

Notably, we obtained 13 statistically significant gene ontology (GO) terms for the proteins containing the G-motifs (*p*-value ≤ 0.05 after multiple testing correction); these relate mainly to organ development, organization and morphogenesis (Supplementary Tables S7, S8). For general Ni-hydrolytic motifs the number of statistically significant GO terms after correction was 145, with 56 related to the mechanisms of regulation of biological processes. We also distinguished a group of 27 GO terms related to the nervous system development, such as regulation of neuron projection development, axon guidance, brain development, neurogenesis and synapse assembly. Further 11 GO terms are related to transcription and gene expression. Detailed classifications of GO terms are provided in Supplementary Tables S9, S10. Overall, the quantitation of occurrence of the Ni-hydrolytic motifs suggests a significant coincidence with the developmental and neuronal functions. Using our database we have also selected immune-related proteins potentially susceptible to Ni-hydrolysis including tumor necrosis factor superfamily, interleukins and interleukin receptors, toll-like receptors and cluster of differentiation proteins (Supplementary Table S11).

The occurrence of hydrolytic motifs in the individual human proteins was compared quantitatively with the expected frequencies of the amino acids (Figures 1F,G). Central portions of those occurrences adhered to the Poisson distributions for both general and G-motifs. However, in both cases there were groups of proteins which substantially deviated from the Poisson distributions, especially those with more than 34 occurrences of the general motifs and 20 occurrences of the G-motifs. Strikingly, proteins with the higher than expected number of these motifs are mostly involved in the epidermal (filaggrin, filaggrin-2, hornerin) and mucosal (mucins) barrier functions (Supplementary Tables S12, S13) which could be important, given that the skin and airway mucosa provide the first line of defence against toxic nickel materials.

### Ni-Hydrolysis Occurs in Filaggrin Model Peptides and the Recombinant Filaggrin Monomer Domain

The barrier proteins abundant in the Ni-hydrolytic motifs are potential targets for Ni^2+^ ions and their concomitant hydrolysis might compromise their function. We chose FLG for the *in vitro* and *ex vivo* experimental verification of this hypothesis due to its largest number of G-motifs (67 in proFLG) and its importance in protection from allergic sensitization, including to nickel (Novak et al., 2008). We also searched our database for other proteins related to keratinocyte differentiation. Interestingly, lorricin, involucrin, trichohyalin and elafin do not contain any hydrolytic motifs, while the previously mentioned filaggrin, filaggrin-2 and hornerin are enriched with such motifs (Supplementary Table S14).

**FIGURE 2:**
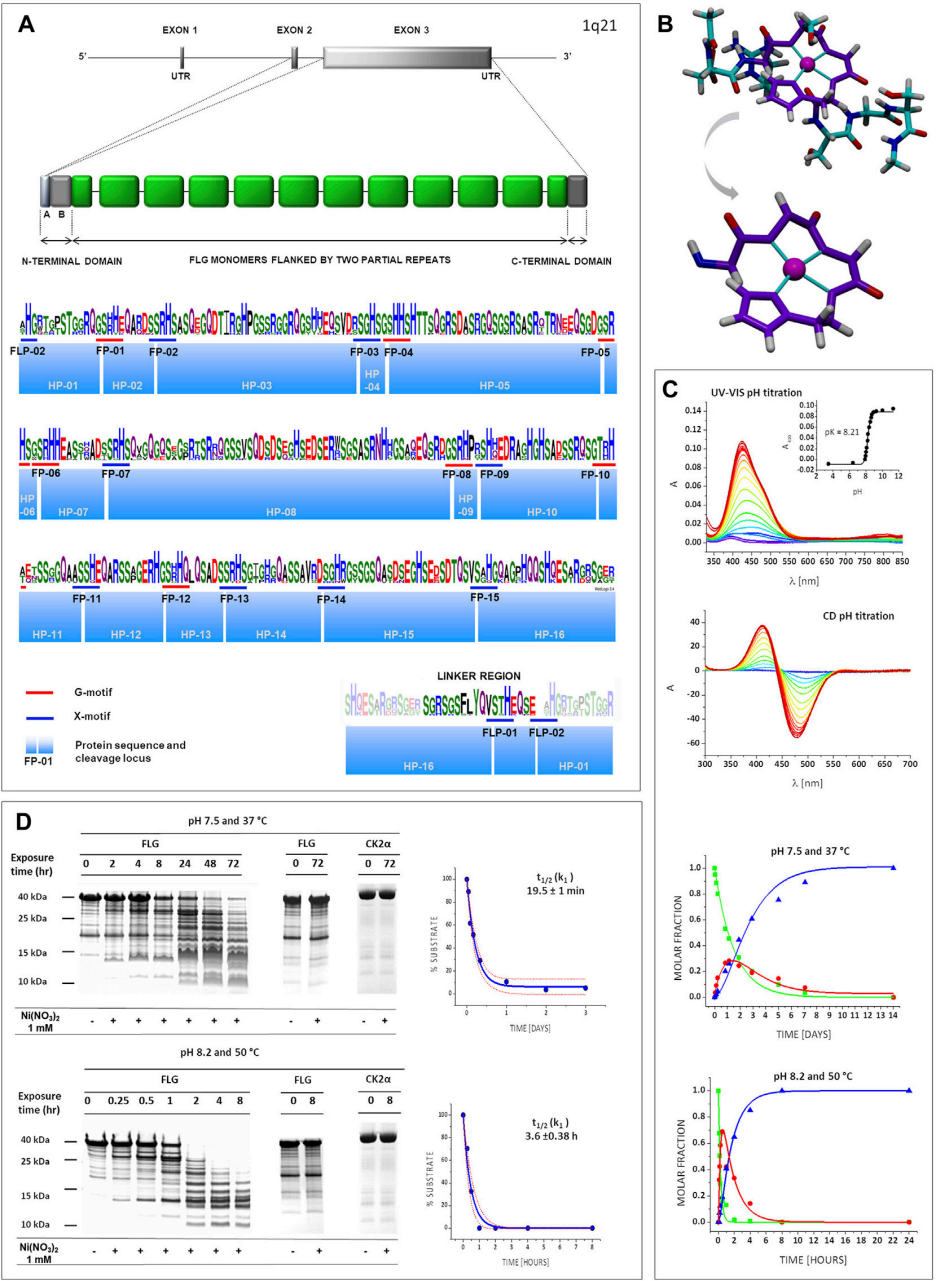
FLG structure and susceptibility to Ni-hydrolysis. **(A)** The *FLG* gene, located on chromosome 1q21, consists of three exons and two introns. ProFLG protein contains several nearly identical FLG monomer units (green) flanked by partial monomer repeats and the N- and a C-terminal domains (dark grey). Each FLG monomer unit is separated from the other repeats by a linker region that is proteolytically cleaved during processing of proFLG into monomers [adapted from Sandilands et al. (2009)]. A graphical representation of an amino acid multiple sequence alignment is shown; the height of a stack indicates the sequence conservation, while the height of symbols within the stack indicates the relative frequency of each amino acid at that position. Colours of amino acids according to chemical properties: polar (green), neutral (purple), basic (blue), acidic (red), hydrophobic (black). Blue and red bars symbolize Ni-hydrolytic motifs; blue boxes symbolize oligopeptides, predicted products of Ni-hydrolysis (HPs). FPs (FLG peptides) and FLP (FLG linker peptide) means consecutive cleavage motifs within the FLG sequence. **(B)** Molecular model of a representative complex (Ni-FP-05). Atoms are marked as: cyan (C), red (O), white (H), indigo (N) and magenta (Ni). 4N square planar structure is highlighted in purple. **(C)** Complexometric and kinetic studies for the Ni-FP-05 complex. CD and UV-VIS pH titration: The pH values marked with colour gradient from dark blue (the lowest pH, 3.5) to red (the highest pH, 11.5). Kinetic studies for the hydrolysis: green squares (substrate), red circles (intermediate product), indigo triangles (final products). **(D)** Ni-hydrolysis of a FLG monomer. Representative gels (left panel) and t_1/2_ (k_1_)graphs (right panel). showing results of cleavage The experimental points are present within the *p* = 0.05 confidence bands, calculated on the basis of the fitted kinetic curve with its standard deviation (red, dotted line).

The FLG monomer domains were identified on the basis of the amino acid sequence of human proFLG, (NCBI database Ref. NP_002007.1) and a prior study on the proFLG component domains (Sandilands et al., 2009). The resulting domains were compared using the WebLogo 3 application. A typical FLG monomer domain contains 17 potential Ni-hydrolytic sites including 7G-motifs with a high degree of conservation, as shown in Figure 2A. Next, we chose eight oligopeptides containing Ni-hydrolytic motifs best representing the cleavage sites, taking into account the variability of third and fifth positions in the X_1_-S/T-c/p-H-c-X_2_ sequence (Supplementary Table S15). The abbreviation numbers of filaggrin peptides (FP) denote the order of their occurrence in the monomer (Figure 2A). Molecular modelling of ten structures of Ni^2+^ complexes with these peptides characterised by the lowest calculated energy levels repeatedly showed square-planar structures with Ni^2+^ chelate ring conformations very similar to each other (Figure 2B, Supplementary Figure S2). Next, comparative CD and UV-vis spectroscopic pH titrations were performed in a broad pH range, yielding the pH dependence of complexation (Figure 2C, Supplementary Figure S3). The calculated pK values showed the absence of stable square-planar complexes below pH 7 for all peptides. Since the Ni^2+^ binding at the individual hydrolytic motifs depends solely on the local sequence, and most of these sites are sufficiently separated from each other, this property of model peptides could be extrapolated over the entire protein (Kopera et al., 2010; Krezel et al., 2010).

Subsequently, we studied the kinetics of FPs hydrolysis as in our previous work (Kopera et al., 2010), using both harsh (50°C, pH 8.2) and physiological (37°C, pH 7.4) conditions. The peptides were hydrolysed in all cases (Figure 2C, Supplementary Figure S4). The kinetic parameters were calculated according to the model of the two sequential first order processes of the intermediate ester formation and decay into final products, as stipulated by the reaction mechanism (Krezel et al., 2010; Podobas et al., 2014). The values of k_1_ and k_2_ rate constants, describing these reaction steps, are presented in Supplementary Table S16. The reaction rates varied depending on the peptide sequence, and the hydrolysis was much faster in harsh conditions, as expected (Kopera et al., 2010).

Next, we confirmed the occurrence of Ni-hydrolysis for the FLG monomer domain, using the recombinant 10th FLG monomer domain (FLG-10, full sequence in Supplementary Table S17). The nickel concentration differed from that used in the peptide model experiments (2 and 1 mM respectively). Nickel hydrolysis has been the subject of extensive investigations in our research group. The conditions chosen for model oligopeptide studies corresponded to previously described experiments on similar peptides (Protas et al., 2013; Podobas et al., 2014; Wezynfeld et al., 2014). The conditions used for FLG domain had lower total Ni^2+^, but higher Ni^2+^/peptide ratio and were aimed at more accurate mirroring of the skin conditions. We would like to note that the Ni^2+^/peptide ratio is more relevant for the reaction rate than the absolute Ni^2+^ concentration, but the rate is ultimately controlled by the cleavage site saturation (Kopera et al., 2010). Recombinant maize protein kinase CK2α which has no Ni-hydrolytic sites (full sequence in Supplementary Table S18) served as a negative control (Figure 2D). Therefore, FLG cleavage resulted specifically from the Ni^2+^ presence rather than a residual protease activity. In order to compare the kinetics of the hydrolysis of FLG-10 vs. the FPs, the rate constants for the latter were recalculated by fitting the first order rate law to the final reaction product formation, as described previously (Krezel et al., 2010) (Supplementary Figure S5 and Supplementary Table S16). This was done since only the final reaction products could be quantified in protein gels, while the separate k_1_ and k_2_ values could be determined for the peptides for an excellent separation of the respective reaction products by HPLC approach (Podobas et al., 2014). The FLG-10 hydrolysis products showed a reproducible pattern of bands, i.e., initially, the two dominant masses (around 25 and 12 kDa) appeared, followed by subsequent hydrolysis of the 25 kDa fragment. The final hydrolysis products had masses within the range of 9–12 kDa, correlating with the cleavage primarily within FP-05 followed by FP-09 and FP-10. As presented in Figure 2D, the t_½_ for the final product formation at harsh conditions (pH 8.2, 50°C) was ca. 20 min vs. 3.6 h at physiological conditions (pH 7.4, 37°C), both reactions proceeded according to the pseudo-first order rate law. The similar time evolution of gel band patterns at these two conditions indicated that the relative reaction rates at individual cleavage sites were maintained in FLG-10. The comparison of fragment sizes at the shortest incubation times with the pattern predicted from the sequence analysis and reactions of peptides confirmed FP-05 and FP-13 as initial reaction sites, followed rapidly by other sites; the entire protein was cleaved into small fragments within hours. Under harsh conditions the rate constant for the FLG domain decay is roughly equal to the sum of rates at the individual hydrolysis sites (Supplementary Figure S6A), while for the physiological conditions the FLG decay is several fold faster than one might expect from the model peptide data Supplementary Figure S6B (note that according to the reaction mechanism the rate constants for cleavages at different FLG sites add up to the overall rate of the domain decay). Altogether, we noticed that at physiological conditions the domain decayed ca. 10 times slower in comparison to the harsh conditions. The multiplicative effect of lowering the reaction temperature and pH on the reaction rate can be estimated as ca. 60–70, stemming from the temperature factor, ca. 2–2.5 and the pH factor, ca. 20–50 (Kopera et al., 2010); here we estimated ca. 40–50 for the most active peptides (Supplementary Figure S6C).

### Ni-Hydrolysis of Filaggrin Occurs in Human Keratinocytes *In Vitro* and in Human Epidermis *Ex Vivo*

**FIGURE 3:**
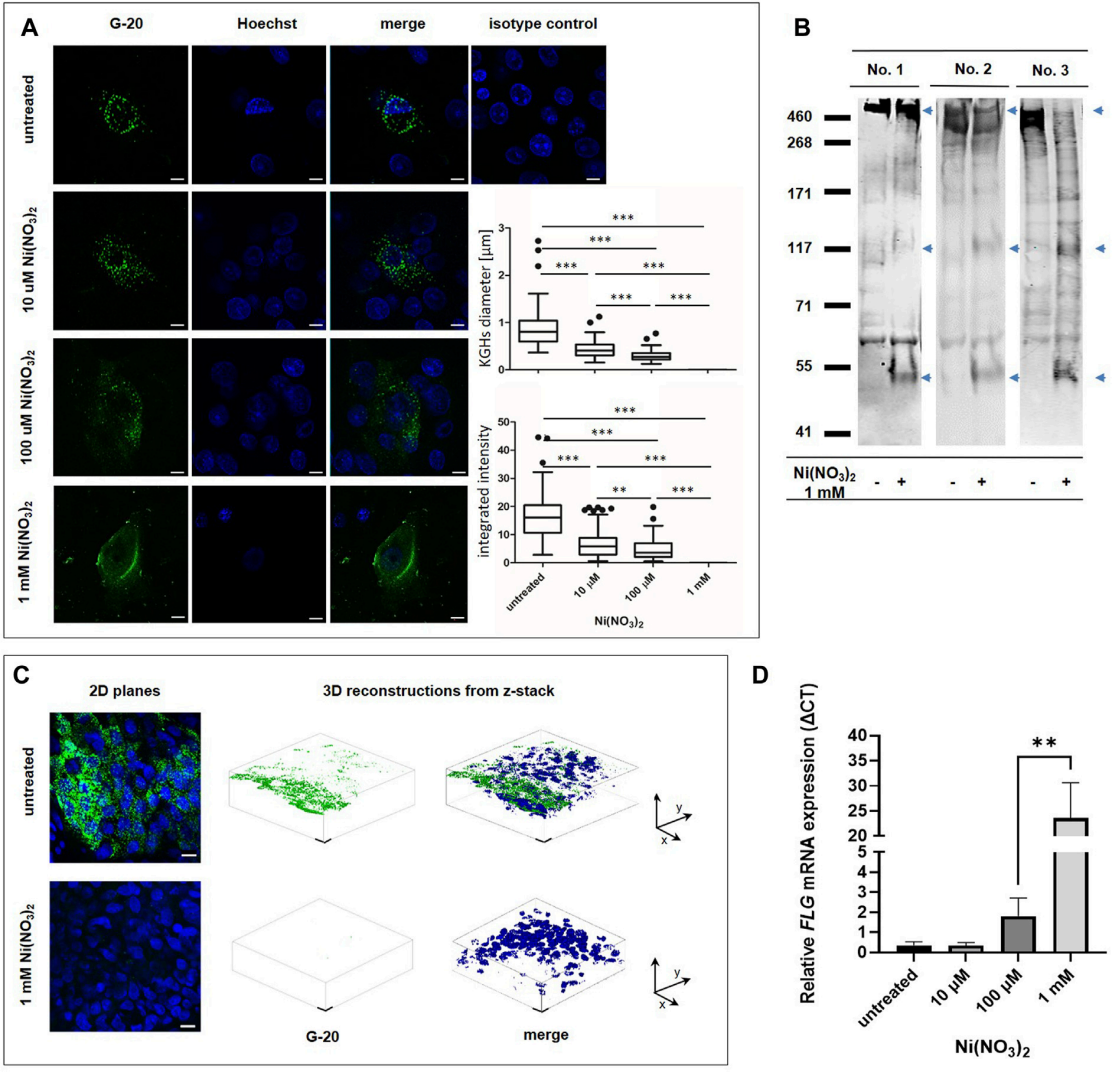
FLG hydrolysis in human keratinocytes *in vitro* and human epidermis *ex vivo*. **(A)** 2D scanning confocal images of fixed NHEK cells immunostained for FLG (green)) and nucleus (blue) after 24 h of treatment with Ni(NO_3_)_2_. Scale bar (10 µM). Diameter of KHGs and integrated intensity from the signal are presented on the Tukey box plots (n∼100 pooled counts from images from 3 independent experiments). In 1 mM nickel concentration it was not possible to observe granules. Error bars represent standard deviation. *p*-value = 0.031 (*t*-test). The main body of the boxplot indicates the interquartile ranges (IQR). Whiskers represent 1.5 x IQR. The median is marked by horizontal lines. Statistical significance is marked with asterisks: (**) *p*-value = 0.0012, (***) *p*-value < 0.0001 (Mann–Whitney *U* test). **(B)** Western blot analysis of FLG level in human epidermal sheets *ex vivo* after treatment 1 mM Ni(NO_3_)_2_ for 36 h, using internal G-20 anti-FLG antibody. Arrows mark the appearance of additional bands due to the cleavage. **(C)** Immunostaining of epidermal sheets with anti-FLG antibodies (green and nucleus (blue). 2D planes and 3D reconstructions from z-stack are presented. Scale bar (10 µM). **(D)** Changes in *FLG* mRNA expression in NHEK cells upon treatment with Ni(NO_3_)_2_ for 24 h. The graph presents ΔCT values with relative to the *TBP1* housekeeping gene expression. Error bars represent standard deviation. *p*-value = 0.031 (*t*-test).

Having determined that the recombinant FLG monomer domain and its model peptides are cleaved by Ni-hydrolysis, we went on to investigate the biological meaning of this phenomenon at both the cellular and tissue levels. Since FLG is expressed predominantly in keratinocytes which are well differentiated, for the cellular study we used normal human epidermal keratinocytes, NHEKs, cultured in the differentiation-promoting medium, i.e. previously well-established calcium-switch model (Gutowska-Owsiak et al., 2018; Gutowska-Owsiak et al., 2020) (Figure 3A). These experiments determined that both the number and sizes of KHGs in NHEKs were reduced upon the Ni^2+^ treatment. Interestingly, some positive staining with anti-FLG antibodies could be observed as KHG-unrestricted cytoplasmic or filamentous signal, suggesting the release of the antibody-reactive FLG-derived peptides into the cytoplasm and potentially binding of those to the intermediate keratin filaments in accordance to the native function of FLG monomers.

To investigate the effect of Ni^2+^ on the abundance of FLG in the stratified epidermis, we used *ex vivo* epidermal sheets obtained from skin samples collected from healthy donors (Figures 3B,C). The exposure to Ni^2+^ resulted in pronounced reduction in the abundance of FLG^+^ KHGs compared to the control samples incubated in the absence of Ni^2+^, as seen in confocal microscope 2D- and Z-stack images. Incubation with 1 mM of Ni^2+^ resulted in a complete disappearance of KGHs. Western blot assessment confirmed this reduction at the protein level in the epidermal sheets exposed to the Ni^2+^ salt. The reduction in the signal coming from the proFLG band (the highest band above 400 kDa) was accompanied by disappearance of the signal of lower molecular weight bands and appearance of unusual bands (marked by arrows on Figure 3B); this was confirmed for the epidermis obtained from three different donors.

The observed reduction in the FLG-related signal was due to the protein degradation and not to the mRNA level suppression, as demonstrated by the quantitative real time PCR performed on NHEKs, where we observed FLG mRNA upregulation with the increasing Ni^2+^ concentration (Figure 3D), likely as a compensatory mechanism.

### Products of Filaggrin Ni-Hydrolysis Affect Langerhans Cells Activation Profile

Finally, we evaluated the impact of Ni-assisted FLG hydrolysis on the phenotype of antigen presenting cells. Here, monocyte derived Langerhans cells (MDLCs) were used to investigate the activation potential of Ni^2+^ complexed to products of hydrolysis of ex-FLG peptides (HP, Figure 2A). Their full amino acid sequences are presented in Supplementary Table S15. For comparative purposes the CD and UV-vis spectroscopic pH titrations of these complexes were performed in a broad pH range. The spectra and titration curves are presented in Supplementary Figure S7. The complex formation process was monophasic, and spectral parameters could be readily assigned to 4N complexes in all cases. In the CD spectra the alternate pattern of d-d bands was observed, typical for the ATCUN motifs (Ariani et al., 2013). All pK values fall in the range of 5.4–5.8, which corresponds to the conditional dissociation constants at pH 7.4 in the range of 1 to 0.1 µM (Sokolowska et al., 2002). We also calculated ten lowest energy structures for all the HPs. The examples of calculated structures of the complexes with Ni^2+^ are presented in Supplementary Figure S8. In every structural variant, the nickel chelate ring conformations with imposed square planar structure are very similar to each other while the N-terminal and C-terminal parts are much more diverse and adopt many conformations in the simulated structures.

MDLCs were incubated with mixed peptides (HP-02, HP-06, HP-07, HP-12, HP-13, HP-14) and the Ni^2+^-complexes (Ni-HPs) formed in molar nickel excess; NiSO_4_ serving as a control; the MDLCs activation was assessed using flow cytometry. In order to gain deeper insights into possible immune pathways that may be affected by the HPs, we also quantified the release of six cytokines from MDLCs, five of which are pro-inflammatory (IFN-α, TNF-α, IL-1β, IL-6, IL-8) and one anti-inflammatory (IL-10). The Luminex assay was performed for HP-06, HP-07 and HP-12.

We noted statistically significant changes between the experimental conditions in results obtained from the same monocyte donor. However, the analysis of pooled results from different biological experiments (between different donors) did not show statistical significance, possibly due to the interindividual variation or relatively low sample number (Supplementary Table S19, S20). Observed trends showed that while the presence of FLG-derived peptides alone did not affect the MDLCs profile in a substantial way, the addition of Ni-HPs resulted in an upregulation of the activation markers (CD86 and HLA-DR) on the cells and a parallel complete loss of the CD80-positivity (Supplementary Figure S9). Ni^2+^ and Ni-HPs conditions are correlated with the increased percentage of CD86^+^ and HLA-DR^+^ cells and loss of the CD80-positivity. As far as the cytokine responses are concerned, we noticed a trend of increased levels of TNFa, IL-6, IL-8 in Ni-HPs in comparison to the nickel only condition (Supplementary Figure S10).

### Analysis of Hydrolytic Motifs Within Filaggrins From Various Species

**FIGURE 4:**
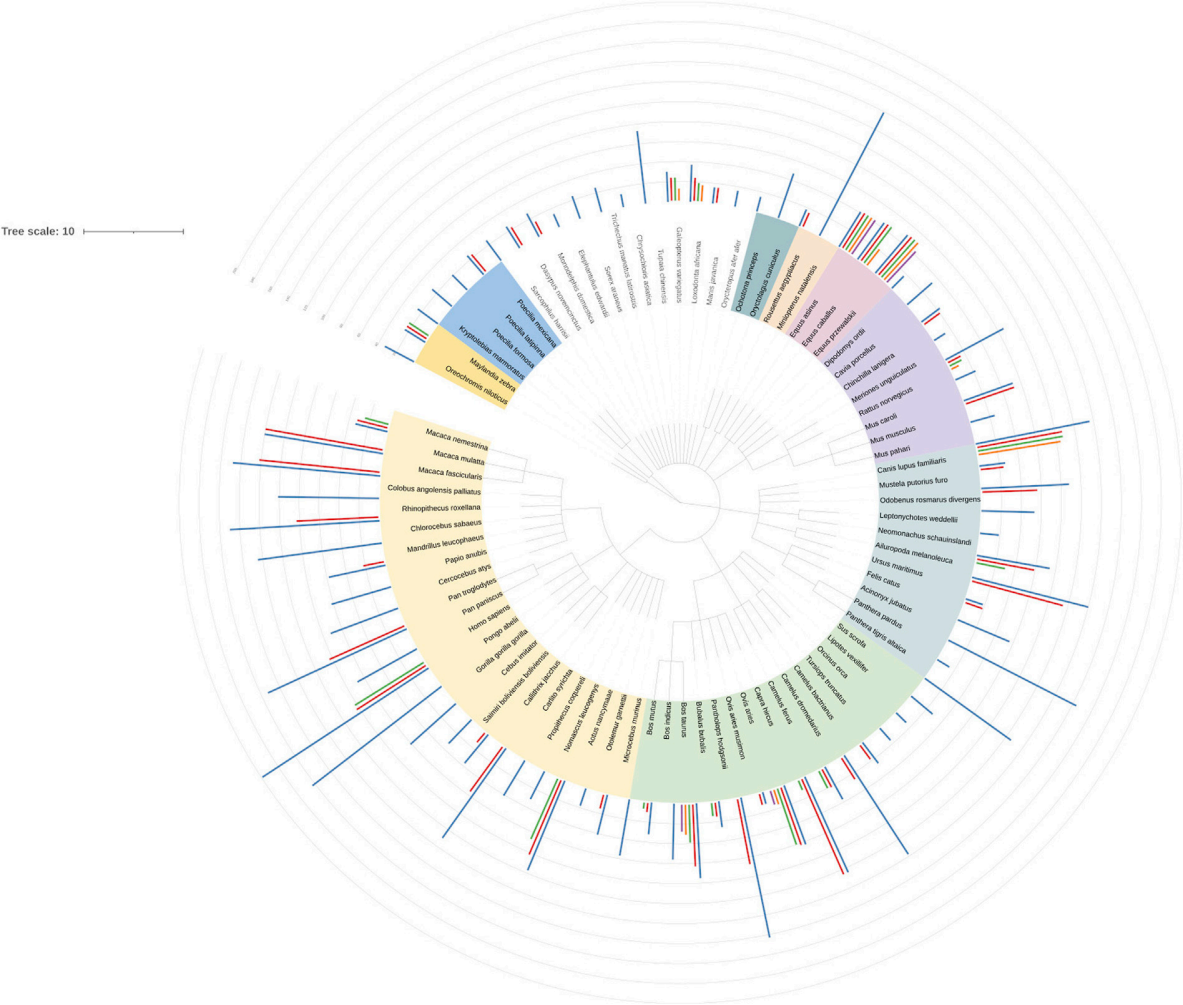
Nickel cleavage sites dataset visualization in iTOL (Letunic and Bork, 2021). Nucleic Acids Res doi: 10.1093/nar/gkab301. Dataset from supplemental table 18 has been used to create a tree in CommonTree (https://www.ncbi.nlm.nih.gov/Taxonomy/CommonTree/wwwcmt.cgi) and visualized in iTOL. Species colours describe taxonomic orders. Data from the ScanProsite analysis of cleavage motifs has been used to create bars outside the tree. Their height indicate the number of cleavage sites.

Comparison of numbers of cleavage motifs between species shows a number of details. The full list of motif counts is presented in Supplementary Table S21 and visualised by the tree of life annotated with hydrolytic motifs datasets (Figure 4). Filaggrin, filaggrin-2 and filaggrin-like proteins were taken into consideration. Analysed species are assigned to the following orders: *Primates* (23), *Artiodactyla* (15), *Carnivora* (11), *Rodentia* (8), *Perissodactyla* (3), *Chiroptera* (2), *Lagomorpha* (2), *Tubulidentata* (1), *Pholidota* (1), *Proboscidea* (1), *Dermopter*a (1), *Scandentia* (1), *Afrosoricida* (1), *Sirenia* (1), *Eulipotyphla* (1), *Macroscelidea* (1), *Didelphimorphia* (1), *Cingulata* (1), *Dasyuromorphia* (1). Filaggrin-like proteins from *Cichliformes* and *Cyprinodontiformes* (*Pisces*) were also included. In all groups of more than 2 species, one can notice differentiation in terms of the number of motifs. In Primates however, filaggrins seem to be enriched; count at least 40 motifs per protein in most cases. Filaggrin in *H.sapiens* is at the top of the list here. Interesting outcome is the startling difference between the number of sites between humans and rodents. Since mice and rats are experimental species this difference shows possible issues when comparing human and rodent data.

## Discussion

The dermal contact with nickel mostly results from surface corrosion of metal objects of daily use by human sweat (Midander et al., 2007). While the dissolved Ni^2+^ ions enter cells adventitiously *via* a divalent metal transporter (DMT) (Chen et al., 2005) or calcium channels (Funakoshi et al., 1997), the highest load results from the phagocytosis of nickel-containing particles, e.g., intracellular concentrations up to 4.75 M Ni^2+^ were calculated for nickel sulphide (Cangul et al., 2002) https://paperpile.com/c/I6dnK0/jWy7l. It is not a typical scenario for skin exposures, but nevertheless possible after contact with nanoparticles from the polluted air in the industrial environment or after contact with nickel particles scratched from the metal surface. Nickel deposition and *stratum corneum* penetration seems to be significant after relatively short exposure (Ahlström et al., 2018). It is known that nickel is accumulated mostly in a bound state in *stratum corneum*; in contrast, only minor concentrations were found below this layer in healthy skin (Fullerton and Hoelgaard, 1988; Hagvall et al., 2021). Ahlström et al. quantified metallic nickel penetration into the *stratum corneum*; nickel deposition was found to be in the range between 10.1 and 23.5 μg/cm^2^ after three 10-min exposures. (Ahlström et al., 2018). Simultaneously range 0.45–12 ug/g of nickel was confirmed in tattooed skin (Kluger, 2021).

The Ni-hydrolysis of specific susceptible protein motifs is a candidate molecular mechanism of nickel allergy and additional adverse effects of nickel exposure in humans (Wezynfeld et al., 2016). We show that such motifs are present in as many as 40% of human proteins. Among them, the glycine-containing motifs are significantly overrepresented.

The gene ontology analysis revealed that proteins containing those Ni-hydrolytic motifs take part in diverse biological processes including transcription and gene expression; thus, we propose that unspecific proteome degradation may lead to a disturbance of cell homeostasis, contributing to both the known mechanisms of nickel toxicity as well as additional underlying adverse processes not yet ascribed to the nickel exposure. Strikingly, at the organism level, FLG and other barrier proteins known for their role in maintaining the integrity of the epidermal barrier and the mucosa, exhibit high incidence of Ni-hydrolytic motifs, making them susceptible to nickel-induced degradation. FLG seems to be especially susceptible, since a single FLG domain contains 17 individual hydrolytic motifs in a repetitive pattern, conserved within all the monomer domains; this results in nearly 200 potential cleavage sites in a single proFLG molecule.

Information about FLG evolution in vertebrate is limited. Comparison nucleotide diversity between FLG repeat regions in primates showed that FLG repeats evolved under the birth-and-death model probably as a consequence of species-specific divergence and expansion (Romero et al., 2017). Skin interacts with the environment what potentially expose it to many adaptive factors. However, because of living in modern and industrialised environment, the character of those factor changed comparing to the natural environment. The purely anthropogenic character of the nickel exposure, related to the industrial revolution and lasting for not more than 250 years (8–10 generations, mine production of nickel began in Norway in 1848) is too short for an evolutionary adaptation to a mildly lethal agent (Kasprzak et al., 2003). The amino acids frequencies within hydrolytic motifs indicate the absence of evolutionary pressures to eliminate them. On the other hand, hydrolytic motifs are present in other non-human species. This suggest that the mutations responsible for the formation of hydrolytic motifs were independent and repetitive. Thus, we should to consider non-anthropogenic factors rather including a possible role of skin microbiome. There has been evidenced of the coevolution between skin microbiota and their corresponding host species (Ross et al., 2018). There are also evidences of the coevolution between skin microbiota and their corresponding host species (Ross et al., 2018). We should not than exclude a possible role of skin microbiome on the filaggrin composition.

Based on our results, we could expect rapid degradation of FLG domains. Interestingly, the hydrolytic sequence present in the inter-domain linker was very poorly reactive, indicating that Ni^2+^ ions would not assist the release of FLG monomers from proFLG with and only the intradomain cleavage resulting in abnormal FLG fragments is likely. The pK values for the complex formation obtained from spectroscopic titrations indicate that hydrolytically productive complexes can form only above pH 7. Due to the locality of Ni^2+^ binding to FLG this feature can be extrapolated over the entire protein. However, the hydrolysis is extremely slow below the pH of 7, as it is enabled by a pH-dependent square-planar Ni^2+^ complex (Kopera et al., 2010). This suggests that in healthy skin, characterised by the surface pH ranging from 4.1 to 5.8 (Segger et al., 2008), Ni-hydrolysis is unlikely. We cannot exclude a possible beneficial effect of the high number of Ni-hydrolysis motifs in FLG. In fact, the repetitive nature of the proFLG structure could possibly point to this. In the case of nickel, there is certainly a strong possibility given the importance of FLG gene null mutations associated with nickel contact sensitization (Novak et al., 2008; Thyssen et al., 2010). It is postulated that FLG chelate Ni^2+^ ions by its numerous histidine side chains and prevent their penetration into deeper layers where interaction with the immune system can promote nickel sensitization (Fullerton and Hoelgaard, 1988; Sigel et al., 1990; Thyssen et al., 2010; Hagvall et al., 2021). It should be first noted that the Ni^2+^-related FLG hydrolytic degradation yields specific oligopeptide complexes in which Ni^2+^ ions are bound more strongly than the original substrate. Moreover, the Ni^2+^ binding should be expected to protect the local sequence S/T-X-H from further proteolysis, by shielding the peptide bonds. Then, the formation of these complexes could potentially facilitate Ni^2+^ penetration of the organism, depending on the properties of these peptides, e.g. the hydrophobicity for membrane penetration. Altogether, the binding of Ni^2+^ to FLG without hydrolysis (possible at low pH) will be protective against Ni^2+^ penetration, but the hydrolysis products may not be as efficient.

However, skin inflammation and keratinocyte differentiation defects lead to a reduction in the content of acidic FLG breakdown products constituting the natural moisturising factor (NMF, i.e., urocanic acid, UA and pyrrolidone carboxylic acid, PCA) within *stratum corneum*. This may result in the elevation of pH up to 9 locally (Schreml et al., 2011) with consequential activation of serine proteases and excessive desquamation. Exposure to Ni^2+^ causing abnormal FLG cleavage could impair NMF generation and further compound the barrier deficiency. On the other hand, Ni-hydrolysis of FLG could also take place intracellularly (pH 7.0–7.4) (Madshus, 1988), as it was demonstrated previously for histone H2A for several cell lines incubated with a NiCl_2_ solution (Karaczyn et al., 2003; Schreml et al., 2011).

We have indeed shown a decrease in KHGs-concentrated proFLG levels compared to controls incubated without Ni(NO_3_)_2_ both at the cellular and tissue levels. However, there are some limitations to this study that could be addressed in future research. First, the work focused on estimating changes in FLG concentration mainly on the basis of immunolocalization and immunodetection techniques. We performed RT-qPCR experiments on *Flg* mRNA levels. This should also be repeated on the epidermal sheets. We could possibly use an additional method to measure the detrimental effect of nickel on the FLG. Quantification of the NMF compartments such as PCA or UCA NMF might be a solution (Koppes et al., 2017). The second limitation that should be discussed here is relatively high nickel concentration used in experiments on the epidermal model. Our aim was to fully saturate all hydrolytic motifs within proFLG with nickel; nickel ions may be chelated by other histidine-rich proteins what might significantly reduce the exact Ni^2+^ concentration and possibly mask the effects of hydrolysis. The IC_50_ value of Ni(NO_3_)_2_ was 1 mM for the monolayer keratinocytes cultures (Supplementary Figure S1). Thus, some not directly related with nickel-hydrolysis toxic effects might occur. However, the data gathered from a Western blot indicated a repetitive pattern of proFLG degradation (Figure 3B). This can be explained by the diverse hydrolysis rates for different motifs. Nevertheless, additional analysis related to FLG monomers in the epidermis would be supplemental.

While the rate constant for the FLG domain decay is roughly equal to the sum of rates at individual hydrolysis sites under harsh reaction conditions, it is several fold higher than expected from these data for the physiological conditions. This can be tentatively interpreted as follows: at harsh conditions all His side chains have lost their positive charges, which may result in the loss of prestructuring of Ni^2+^ binding sites enabled by ionic interactions and H-bonds. Such prestructuring was shown to accelerate the hydrolysis (Wezynfeld et al., 2014), but is absent from short model peptides studied here, thus explaining the 5-fold acceleration of FLG hydrolysis at physiological conditions over the expectations.

The studied process yields C-terminal reaction products of FLG cleavage in the Ni^2+^-complexed form. Dissociation constants of these complexes at pH 7.4 are in the range of 0.1–1 μM (Sokolowska et al., 2002); these complexes are slow to release Ni^2+^ ions by dissociation even if the pH is decreased or the complex gets diluted in the body fluids or makes contact with a stronger chelator (Bal et al., 1996; Sokolowska et al., 2002). This makes them potential candidate Ni^2+^ carriers. A hypothetical protein playing a similar role and activating antigen presenting cells was proposed previously in the literature (Thierse et al., 2004, 2005) The synthetic peptides modelling the products of Ni-hydrolysis might thus be used as a potential tool in nickel allergy research.

In this context, we may propose that the similarity of effects on dendritic cells between free vs complexed Ni^2+^ results from the ability of added Ni^2+^ ions to recruit ligands in the vicinity or on the cell surface which may provide chemical environment for Ni^2+^ similar to that present in FLG peptides. Not only the abundance of such “*prêt-à-porter*” ligands in the extracellular space of the skin may be high, e.g., the serum albumin is present in the extracellular fluid at sub millimolar concentrations. The formation of Ni^2+^ 4N complex with this sequence is a spontaneous process that takes about an hour at neutral pH (Bal et al., 1998). Ni^2+^-albumin complexes were previously shown to stimulate Ni-reactive T cells in the presence of antigen presenting cells (Thierse et al., 2005) which may partially explain the activation of MDDCs exposed to nickel control in our experiments.

On the other hand, the presence of 50 µM Ni^2+^-complexed HPs seemingly caused a stronger effect, which should be however confirmed with more biological replicates. Furthermore, it is important to stress that the database of human proteins highlighted many more targets potentially susceptible to Ni-hydrolysis, including some immune-related. Those include cytokines produced by dendritic cells upon the Ni^2+^ exposure (such TNF-α, IL-6), cluster of differentiation markers playing an important role in T-cell activation (CD80, CD86) and innate recognition receptors such as TLRs; these induce proinflammatory cytokine production and antigen presentation to T-cells. Thus, Ni-hydrolysis might dysregulate important immune responses. On the other hand we cannot exclude a possibility of spontaneous nickel-assisted degradation of those protein compartments similar to that observed for FLG; this factor could hinder the interpretation and should be taken into account. An interesting extension of our research work would be to study cytokine levels after keratinocyte exposure to nickel. Keratinocytes may act as instigators of cutaneous inflammation (Gutowska-Owsiak and Ogg, 2012) through inflammatory cytokine secretion, having an influence on Langerhans cell migration to the draining lymph nodes and T cell trafficking (Barker et al., 1991; Nestle et al., 2009). On the other hand, elevated levels of inflammation markers after keratinocyte exposure to nickel were already reported (Sainte-Marie et al., 1998).

The results presented above indicate that Ni^2+^ ions can cause FLG degradation *via* direct, non-enzymatic hydrolysis within minutes, and suggest that the hydrolysis products may trigger activation of Langerhans cells with accompanied proinflammatory *milieu* in the skin during nickel contact skin allergy. Indeed, Ni^2+^ ions have been previously shown to modulate intracellular pathways in dendritic cells via NF-κB activity and p38 MAPK regulation (Boisleve et al., 2005; Ade et al., 2007). Ni^2+^ was also documented as a regulator of the IL-12 production, important in Th1-driven immune responses (Antonios et al., 2010). Moreover, NiSO_4_ was already shown to induce the expression of HLA-DR, CD83, CD86, and CD40 and production of IL-8, IL-6, and IL-12p40 in human dendritic cells (Ade et al., 2007).

Proteomic studies with human monocytes identified protein species linked to distinct molecular processes including cell death, that are specifically regulated by Ni^2+^; the regulation mechanism was not clarified (Jakob et al., 2017). Interestingly, almost half of the aforementioned proteins contain the Ni^2+^ hydrolytic motifs; underscoring the plausibility of the mechanism identified in this study. These findings are applicable to the known occupational hazards of inhalatory nickel exposure (Kasprzak et al., 2003), and to postulated relevance of lower-level exposure of the general population to nickel present in particles generated by combustion of fossil fuels, tobacco smoke and corrosion of metal objects (Vouk and Piver, 1983; Pappas, 2011). Specifically, small particles suspended in the polluted air appear to be particularly toxic; those sized 2.5 µm or less (PM 2.5) and classified as carcinogens by IARC (IARC Working Group on the Evaluation of Carcinogenic Risks to Humans and International Agency for Research on Cancer, 2016) can penetrate through the alveolar epithelium and enter the bloodstream, leading to the secondary tissue accumulation (Li et al., 2015) and, possibly, induction of inflammation (Wang et al., 2017). Recently, the smallest metal nanoparticles were also found to penetrate into the brain via the olfactory bulb (Tallkvist et al., 1998; Oberdörster et al., 2009; Maher et al., 2016); carrying a danger of Ni-hydrolysis directly in the brain. In summary, our results propose a novel, broadly applicable mechanism which could contribute to multiple known nickel-related pathologies and could help identify the relationships of nickel exposure with additional toxic effects.

Ni^2+^-assisted cleavage of barrier proteins, including FLG, may contribute to clinical disease associated with nickel exposure.

## Data Availability

The original contributions presented in the study are included in the article/Supplementary Material, further inquiries can be directed to the corresponding authors.
