# Corrigendum: Ni^2+^-assisted hydrolysis may affect the human proteome; filaggrin degradation *ex vivo* as an example of possible consequences

**DOI:** 10.3389/fmolb.2022.1101224

**Published:** 2022-12-12

**Authors:** Ewa Izabela Podobas, Danuta Gutowska-Owsiak, Sébastien Moretti, Jarosław Poznański, Mariusz Kulińczak, Marcin Grynberg, Aleksandra Gruca, Arkadiusz Bonna, Dawid Płonka, Tomasz Frączyk, Graham Ogg, Wojciech Bal

**Affiliations:** ^1^ Institute of Biochemistry and Biophysics, Polish Academy of Sciences, Warsaw, Poland; ^2^ Medical Research Council Human Immunology Unit, National Institute for Health Research Oxford Biomedical Research Centre, Medical Research Council Weatherall Institute of Molecular Medicine, University of Oxford, Oxford, United Kingdom; ^3^ Institute of Genetics and Biotechnology, University of Warsaw, Warsaw, Poland; ^4^ University of Gdansk, Intercollegiate Faculty of Biotechnology of University of Gdańsk and Medical University of Gdańsk, Gdańsk, Poland; ^5^ SIB Swiss Institute of Bioinformatics, Vital-IT Team, Lausanne, Switzerland; ^6^ The Maria Sklodowska-Curie National Research Institute of Oncology, Warsaw, Poland; ^7^ Institute of Informatics, Faculty of Automatic Control, Electronics and Computer Science, Silesian University of Technology, Gliwice, Poland; ^8^ Department of Biochemistry, University of Cambridge, Cambridge, United Kingdom

**Keywords:** filaggrin, human proteome, protein degradation, Ni^2+^-assisted hydrolysis, nickel toxicity, nickel allergy

In the published article, there was an error in the author list, and author Tomasz Frączyk was erroneously excluded. Furthermore, an author’s initials were incorrectly written as AD in the **Author Contribution** section. The correct spelling is AG. A correction has therefore been made to the **Author Contribution** statement. It previously stated:

“This project was conceived and designed by EIP, DG-O, MG and WB. GO supervised the work. All of the experimental work was performed by EIP, DG-O, MK, AB, and DP. Computational analyses were performed by SM, AD, and JP. The manuscript was written and prepared by EIP, DG-O and WB”.

The corrected statement appears below:

“This project was conceived and designed by EIP, DG-O, MG, and WB. GO supervised the work. All of the experimental work was performed by EIP, DG-O, MK, AB, and DP. Selection of some FLG model peptides (QAASSHEQA, YQVSTHEQS, ADSSRHSGI) at the initial stage of the project was performed by TF. Computational analyses were performed by SM, AG, and JP. The manuscript was written and prepared by EIP, DG-O, and WB”.

The authors apologize for these errors and state that this does not change the scientific conclusions of the article in any way. The original article has been updated.

