# Supplementary material for: Ni2+-Assisted Hydrolysis May Affect the Human Proteome; Filaggrin Degradation Ex Vivo as an Example of Possible Consequences
Source: Front Mol Biosci. 2022 Mar 10;9:828674. doi: 10.3389/fmolb.2022.828674 (PMC8960189; doi:10.3389/fmolb.2022.828674)
Supplement: Supplementary file 1 [file DataSheet2.pdf]

Supplementary Material

TABLE OF CONTENTS

1 Supplementary Tables.…………………..………………………………………… 2

2 Supplementary Figures..……………………………………………………….... 13

3 Supplementary References…….………………………………………………….. 27

1 Supplementary Tables

Supplementary Table 1 (Supplementary Excel file)
List of human proteins contained X_1-S/T-c/p-H-c-X_2

Supplementary Table 2 (Supplementary Excel file)
List of human proteins contained G-S/T-c/p-H-c-X_2

Supplementary Table 3 (Supplementary Excel file)
List of human proteins with no identified X_1-S/T-c/p-H-c-X_2

Supplementary Table 4 (Supplementary Excel file)
List of human proteins with no identified G-S/T-c/p-H-c-X_2

Supplementary Table 5 (Supplementary Excel file)
List of X_1-S/T-c/p-H-c-X_2

Supplementary Table 6 (Supplementary Excel file)
List of G-S/T-c/p-H-c-X_2

Supplementary Table 7 (Supplementary Excel file)
The full list of statistically significant GO terms for the X_1-S/T-c/p-H-c-X_2 -containing proteins

Supplementary Table 8 (Supplementary Excel file)
The full list of statistically significant GO terms for G-S/T-c/p-H-c-X_2 -containing proteins

Supplementary Table 9

Functional groups for statistically significant GO terms for the G-S/T-c/p-H-c-X_2 -motifs-containing proteins

Functional group description	GO terms
**GO terms related to organs development, organization and morphogenesis**	nervous system development; multicellular organism development; anatomical structure development; anatomical structure morphogenesis; developmental process; cell morphogenesis; cellular component morphogenesis; cytoskeleton organization; system development; organelle organization
**GO terms related to metabolic and biosynthesis**	one-carbon metabolic process; leukotriene D4 metabolic process; leukotriene D4 biosynthetic process

Supplementary Table 10

Functional groups for statistically significant GO terms for X_1-S/T-c/p-H-c-X_2 -motifs-containing proteins

Functional group description	GO terms
**GO terms related to regulation of biological processes**	regulation of transcription by RNA polymerase II; regulation of macromolecule biosynthetic process; regulation of cellular macromolecule biosynthetic process; regulation of RNA metabolic process; regulation of nucleic acid-templated transcription; regulation of RNA biosynthetic process; regulation of transcription, DNA-templated; regulation of nucleobase-containing compound metabolic process; regulation of cellular biosynthetic process; regulation of biosynthetic process; regulation of cellular process; biological regulation; regulation of cellular metabolic process; regulation of biological process; regulation of primary metabolic process; regulation of gene expression; regulation of nitrogen compound metabolic process; regulation of macromolecule metabolic process; regulation of nervous system development; regulation of cell development; regulation of metabolic process; regulation of neurogenesis; regulation of small GTPase mediated signal transduction; regulation of GTPase activity; regulation of cellular component organization; positive regulation of RNA metabolic process; positive regulation of RNA biosynthetic process; positive regulation of transcription, DNA-templated; positive regulation of nucleic acid-templated transcription; positive regulation of nervous system development; regulation of developmental process; regulation of cell morphogenesis; positive regulation of GTPase activity; regulation of multicellular organismal development; positive regulation of cell development; regulation of neuron projection development; positive regulation of nucleobase-containing compound metabolic process; regulation of Ras

	protein signal transduction; positive regulation of macromolecule biosynthetic process; regulation of multicellular organismal process; positive regulation of transcription by RNA polymerase II; regulation of neuron differentiation; regulation of cell differentiation; regulation of anatomical structure morphogenesis; positive regulation of neurogenesis; positive regulation of developmental process; positive regulation of cellular metabolic process; positive regulation of macromolecule metabolic process; positive regulation of cellular component organization; regulation of Rho protein signal transduction; regulation of cell projection organization; regulation of plasma membrane bounded cell projection organization; positive regulation of cell differentiation; regulation of organelle organization; regulation of axonogenesis; positive regulation of nitrogen compound metabolic process;
**GO terms related to nervous system development**	nervous system development; regulation of nervous system development; neurogenesis; generation of neurons; regulation of neurogenesis; neuron development; neuron projection morphogenesis; neuron differentiation; positive regulation of nervous system development; neuron projection development; cell morphogenesis involved in neuron differentiation; axon development; central nervous system development; synapse organization; regulation of neuron projection development; axonogenesis; head development; brain development; neuron projection guidance; axon guidance; regulation of neuron differentiation; neuron projection extension; positive regulation of neurogenesis; synapse assembly; forebrain development; synaptic transmission, glutamatergic; regulation of axonogenesis
**GO terms related to transcription and gene expression**	regulation of transcription by RNA polymerase II; transcription by RNA polymerase II; regulation of nucleic acid-templated transcription; regulation of transcription, DNA-templated; nucleic acid-templated transcription; transcription, DNA-templated; positive regulation of transcription, DNA-templated; positive regulation of nucleic acid-templated transcription; positive regulation of transcription by RNA polymerase II; regulation of gene expression; gene expression

Supplementary Table 11

Selected human proteins related to immune system containing nickel-assisted cleavage motifs.

Name	Function
FOXP 3	FOX protein family
TNFA, TNFB	Tumor necrosis factor superfamily
IL1B, IL3, IL4, IL5, IL6, IL13, IL15, IL16, IL17F, IL23A, IL24, IL27A, IL27B, IL28A, IL29, IL32, IL33, IL36B, IL37	Interleukins
IL1R1, IL1R2, IL2RB, IL2RA, IL3RA, IL4RA, IL5RA, IL6RA, IL7RA, IL23R, IL31R	Interleukin receptors
TLR1, TLR2, TLR3, TLR4, TLR5, TLR6, TLR7, TLR8, TLR9, TLR10	Toll-like receptors
CD1A, CD1B, CD1C, CD1E, CD2, CD6, CD7, CD8B, CD11A, CD19, CD27, CD28, CD34, CD38, CD44, CD80, CD81, CD86, CD109, CD158, D166, D177, CD180, CD181, D248, CD302	Cluster of differentiation proteins

Supplementary Table 12

Full list of proteins substantially deviated from Poisson distributions in quantitative analysis of occurrence of hydrolytic motifs per protein compared with the expected frequencies of amino acids. Results for X_1-S/T-c/p-H-c-X_2 -motifs-containing proteins.

**UniProt AC**	**Protein length**	**Motifs number**	**Protein name**
A0A0G2JRJ6	6464	186	Mucin-4
A0A0G2JRY3	6450	186	Mucin-4
P20930	4061	169	Filaggrin
Q9UKN1	5478	150	Mucin-12
A0A0G2JM16	5314	117	Mucin-4
Q8WXI7	14507	85	Mucin-16
Q86YZ3	2850	85	Hornerin
Q5D862	2391	73	Filaggrin-2
Q8WZ42-12	35991	68	Isoform 12 of Titin
Q8WZ42-8	34475	67	Isoform 8 of Titin
D3DPG0	34942	66	Titin, isoform CRA_a
Q8WZ42-13	34484	66	Isoform 13 of Titin
Q8WZ42	34350	66	Titin
Q8WZ42-7	33615	65	Isoform 7 of Titin
Q8WZ42-5	32900	65	Isoform 5 of Titin
Q8WZ42-4	33445	64	Isoform 4 of Titin
Q8WZ42-11	33423	64	Isoform 11 of Titin
Q8WZ42-9	27118	49	Isoform 9 of Titin
Q8WZ42-10	27051	49	Isoform 10 of Titin
Q8WZ42-3	26926	48	Isoform 3 of Titin
A7Y9J9	6207	47	Mucin 5AC, oligomeric mucus/gel-forming
Q9HC84	5762	45	Mucin-5B
Q02505	3323	43	Mucin-3A
Q02505-5	3265	43	Isoform 5 of Mucin-3A
Q2LD37	5005	35	Transmembrane protein KIAA1109
Q6W4X9	2439	35	Mucin-6

Supplementary Table 13

Full list of proteins substantially deviated from Poisson distributions in quantitative analysis of occurrence of hydrolytic motifs per protein compared with the expected frequencies of amino acids. Results for the G-S/T-c/p-H-c-X_2 -motifs-containing proteins.

UniProt AC	Protein length	Motifs number	Protein name
P20930	4061	67	Filaggrin
Q86YZ3	2850	34	Hornerin
Q5D862	2391	24	Filaggrin-2
Q9UKN1	5478	22	Mucin-12
A7Y9J9	6207	21	Mucin 5AC, oligomeric mucus/gel-forming
Q9HC84	5762	21	Mucin-5B

Supplementary Table 14

Selected human proteins related to keratinocyte differentiation.

UniProt AC	Protein length	Motifs number	Protein name
P20930	4061	169	Filaggrin
Q5D862	2391	73	Filaggrin-2
Q86YZ3	2850	85	Hornerin
P04264	644	1	Keratin, type II cytoskeletal 1
P13645	584	1	Keratin, type I cytoskeletal 10
P07476	585	0	Involucrin
P23490	312	0	Loricrin
Q07283	1943	0	Trichohyalin
P19957	117	0	Elafin

Supplementary Table 15

Sequences of synthesised 9 amino acid long filaggrin oligopeptides (FPs, FLP) and hydrolysis products (HPs). Ac- denotes the N-terminal acetylation, and -am the C-terminal amidation of the peptide chain. Cleavage motifs in bold.

	Abbreviation	Sequence
**Filaggrin peptides**	**FP-02**	Ac-RD**SSRHS**AS-am
	**FP-05**	Ac-GD**GSRHS**GS-am
	**FP-09**	Ac-HP**RSHHE**DR-am
	**FP-10**	Ac-SG**GTRHA**ET-am
	**FP-11**	Ac-QA**ASSHE**QA-am
	**FP-13**	Ac-AD**SSRHS**GI-am
	**FP-14**	Ac-VR**DSGHR**GS-am
	**FLP-01**	Ac-YQ**VSTHE**QS-am
**Hydrolysis products**	**HP-02**	-SHHEQARDS-
	**HP-06**	-SRHSG-
	**HP-07**	-SRHHEASSADS-
	**HP-10**	-SSHEQARSSAGERHG-
	**HP-12**	-SRHSGIGHGQASSAVRD-
	**HP-13**	-SGHRGYSGSQASDNEGHSEDSDTQSV-
	**HP-14**	-SAHGQAGSHQQSHQESARGRSGET-

Supplementary Table 16

The $t_{1/2}(k_1)$ and $t_{1/2}(k_2)$ values for the two-step sequential hydrolysis of filaggrin peptides (FPs) and FLG recombinant domain (FLG-10) at physiological and harsh conditions in the presence of Ni^{2+}.

	pH 7.4, 37 °C			pH 8.2, 50 °C		
**Peptide**	$t_{1/2}$ (k_1) [h] ± S.D.	$t_{1/2}$ (k_2) [h] ± S.D.	$t_{1/2}$ (k) [h] ± S.D.	$t_{1/2}$ (k_1) [min] ± S.D.	$t_{1/2}$ (k_2) [min] ± S.D.	$t_{1/2}$ (k) [min] ± S.D.
FP-02	42 ± 3	19.5 ± 2	72 ± 4	35 ± 1.7	38 ± 4.5	93 ± 9
FP-05	25 ± 1.5	22.5 ± 4	55.7 ± 1.5	10.5 ± 1	65 ± 4.5	82.3 ± 5
FP-09	40 ± 1	1 ± 0.2	41 ± 1	40 ± 4.5	11 ± 3.5	62.8 ± 7
FP-10	57.5 ± 6	37.2 ± 7.7	106 ± 5.5	33 ± 5.6	218.5 ± 22	343 ± 19.2
FP-11	105 ± 4	26.8 ± 3.7	154.1 ± 8.3	19.3 ± 1	136 ± 8	173.3 ± 9.4
FP-13	7.4 ± 1.7	50.6 ± 4.7	68.5 ± 4.2	26.5 ± 1.5	52 ± 2	97.7 ± 9.3
FP-14	95 ± 7.7	26.5 ± 6.5	139.4 ± 6	196 ± 9	262.5 ± 9.5	777.3 ± 55.6
FLP-01	3308 ± 246	652 ± 65	3157 ± 389	476 ± 43	1234 ± 44	21708 ± 205
FLG-10	N/A	N/A	3.6 ± 0.38	N/A	N/A	19.5 ± 1

Supplementary Table 17

Sequence and physiochemical properties of recombinant filaggrin monomer FLG-10. Information on the base of ExPASy ProtParam tool (Wilkins et al., 1999). Nickel binding loci are marked: red - strong (micromolar or stronger) including the His-tag, yellow - intermediate (micro to millimolar), green - weak (millimolar), (Bal et al., 1995, 1998; Sunderman et al., 1996; Kozłowski et al., 1999; Mylonas et al., 2002; Kopera et al., 2010).

FLG-10 MONOMER AMINOACID SEQUENCE

10	20	30	40	50	60
AHGRTRTSTG	RRQGSHHEQA	RDSSRHSASQ	EGQDTIRGHP	GSSRRGRQGS	HYEQSVDRSG
70	80	90	100	110	120
HSGSHHSHTT	SQGRSDASRG	QSGSRSASRQ	TRNDEQSGDG	SRHSWSHHHE	ASTQADSSRH
130	140	150	160	170	180
SQSGQGQSAG	PRTSRNQGSS	VSQDSDSQGH	SEDSERWSGS	ASRNHRGSAQ	EQSRDGSRHP
190	200	210	220	230	240
TSHHEDRAGH	GHSAESSRQS	GTHHAENSSG	GQAASSHEQA	RSSAGERHGS	HHQQSADSSR
250	260	270	280	290	300
HSGIGHGQAS	SAVRDSGHRG	SSGSQASDSE	GHSEDSDTQS	VSAHGQAGPH	QQSHQESTRG
310					
RSAGRHHHHH H					

FLG-10 AMINOACID COMPOSITION (number of residues and percentage)

Ala (A) 24 7.7%	His (H) 41 13.2%	Ser (S) 76 24.4%
Arg (R) 35 11.3%	Ile (I) 2 0.6%	Thr (T) 13 4.2%
Asn (N) 4 1.3%	Leu (L) 0 0.0%	Trp (W) 2 0.6%
Asp (D) 17 5.5%	Lys (K) 0 0.0%	Tyr (Y) 1 0.3%
Cys (C) 0 0.0%	Met (M) 0 0.0%	Val (V) 4 1.3%
Gln (Q) 31 10.0%	Phe (F) 0 0.0%	Pyl (O) 0 0.0%
Glu (E) 16 5.1%	Pro (P) 4 1.3%	Sec (U) 0 0.0%
Gly (G) 41 13.2%		

Supplementary Table 18

Sequence and physiochemical properties of maize protein kinase CK2α. Information on the base of ExPASy ProtParam tool (Wilkins et al., 1999).

MAIZE PROTEIN KINASE CK2α AMINOACID SEQUENCE

```
        10          20          30          40          50          60
MSKARVYTDV  NVLRPKEYWD  YEALTVQWGE  QDNYEVVRKV  GRGKYSEVFE  GINVNNNEKC

        70          80          90         100         110         120
IIKILKPVKK  KKIKREIKIL  QNLYGGPNIV  KLLDIVRDQH  SKTPSLIFEY  VNNTDFKVLY

       130         140         150         160         170         180
PTLTDYDIRY  YIYELLKALD  YCHSQGIMHR  DVKPHNVMID  HELRKLRLID  WGLAEFYHPG

       190         200         210         220         230         240
KEYNVRVASR  YFKGPELLVD  LQDYDYSLDM  WSLGCMFAGM  IFRKEPFFYG  HDNHDQLVKI

       250         260         270         280         290         300
AKVLGTDGLN  VYLNKYRIEL  DPQLEALVGR  HSRKPWTKFI  NADNQHLVSH  EAIDFLDKLL

       310         320         330         340
RYDHQDRLTA  REAMTHPYFQ  QVRAAENSTT  RALEHHHHHH
```

CK2α AMINOACID COMPOSITION (number of residues and percentage)

Ala (A) 15 4.4%	His (H) 19 5.6%	Ser (S) 11 3.2%
Arg (R) 21 6.2%	Ile (I) 20 5.9%	Thr (T) 12 3.5%
Asn (N) 18 5.3%	Leu (L) 35 10.3%	Trp (W) 5 1.5%
Asp (D) 25 7.4%	Lys (K) 28 8.2%	Tyr (Y) 23 6.8%
Cys (C) 3 0.9%	Met (M) 7 2.1%	Val (V) 25 7.4%
Gln (Q) 12 3.5%	Phe (F) 12 3.5%	Pyl (O) 0 0.0%
Glu (E) 21 6.2%	Pro (P) 12 3.5%	Sec (U) 0 0.0%
Gly (G) 16 4.7%		

Supplementary Table 19

P-values for data presented in the **Supplementary Figure 9**. Data from three separate biological experiments (n=3). Bonferroni-adjusted significance tests for pairwise comparisons was used to calculate adjusted p-value (**α:** 0.00833).

		**untreated vs nickel**	**untreated vs peptide**	**untreated vs complex**	**nickel vs peptide**	**nickel vs complex**	**peptide vs complex**
CD86 P6	P-value	0.56598625	0.95304113	0.46247547	0.58661172	0.90463371	0.48076068
CD86 P7	P-value	0.56598625	0.86211797	0.44782902	0.50421551	0.87309191	0.39641527
CD86 P12	P-value	0.45554238	0.96611841	0.20231835	0.44374509	0.55932974	0.20418122
CD80 P6	P-value	0.38717817	0.94873474	0.38536766	0.35117555	0.95763101	0.35007242
CD80 P7	P-value	0.28899838	0.86351309	0.28496305	0.27727165	0.94222774	0.27397528
CD80 P12	P-value	0.28899838	0.78235834	0.2893318	0.29200703	0.98985682	0.29228077
HLA DR P6	P-value	0.50046395	0.94636669	0.38816146	0.50956378	0.93518638	0.39841649
HLA DR P7	P-value	0.16080601	0.94547333	0.1302133	0.16729864	0.95469328	0.13623613
HLA DR P12	P-value	0.11089416	0.71865856	0.00784199	0.12231485	0.83629732	0.01087941

Supplementary Table 20

P-values for data presented in the **Supplementary Figure 10**. Data from three separate biological experiments (n=3). Bonferroni-adjusted significance tests for pairwise comparisons was used to calculate adjusted p-value (**α:** 0.00833).

		**untreated vs nickel**	**untreated vs peptide**	**untreated vs complex**	**nickel vs peptide**	**nickel vs complex**	**peptide vs complex**
IFNa2 P6	P-value	0.268469565	0.662502014	0.394034051	0.208658296	0.778275578	0.337657922
IFNa2 P7	P-value	0.268469565	0.566980661	0.46846259	0.187113855	0.937003226	0.381553983
IFNa2 P12	P-value	0.268469565	0.674163949	0.544092154	0.213246395	0.583769086	0.41783495
IL-10 P6	P-value	0.642005625	0.400580786	0.630147828	0.378177608	0.930268725	0.429380551
IL-10 P7	P-value	0.642005625	0.390366839	0.592211532	0.373487157	0.849769825	0.423444262
IL-10 P12	P-value	0.642005625	0.928138869	0.460440281	0.602527403	0.404435566	0.518346614
IL-1b P6	P-value	0.383078453	0.942310103	0.527238026	0.496844783	0.663517858	0.598692691
IL-1b P7	P-value	0.383078453	0.969056099	0.65817998	0.474520615	0.621116418	0.683047074
IL-1b P12	P-value	0.383078453	0.859456471	0.297906659	0.403994636	0.550681516	0.344753925
IL-6 P6	P-value	0.371346068	0.883028292	0.362270735	0.384117303	0.917594611	0.371972687
IL-6 P7	P-value	0.371346068	0.985333578	0.389858265	0.370186849	0.669131134	0.389209512
IL-6 P12	P-value	0.371346068	0.973151892	0.670702344	0.37478802	0.420146109	0.707587727
IL-8 P6	P-value	0.306852867	0.981726867	0.187435909	0.444598346	0.657275738	0.31112442
IL-8 P7	P-value	0.306852867	0.820018074	0.182998773	0.252611701	0.647864683	0.162019678
IL-8 P12	P-value	0.306852867	0.947296782	0.210599787	0.273869292	0.706766128	0.192070194
TNF-a P6	P-value	0.213723977	0.439625043	0.252107932	0.190555823	0.929965103	0.232019655
TNF-a P7	P-value	0.213723977	0.295169557	0.335514189	0.184491142	0.613811153	0.318764265
TNF-a P12	P-value	0.213723977	0.621953429	0.084098384	0.195697006	0.538427543	0.067921175

Supplementary Table 21 (Supplementary Excel file)

Analysis of hydrolytic motifs within filaggrins from various species.

2 Supplementary Figures

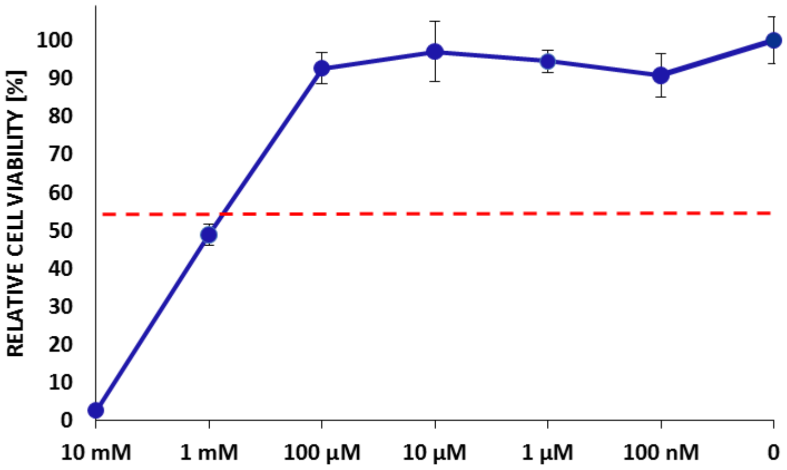

Supplementary Figure 1. The cell proliferation assay (MTT) for keratinocytes exposed to $Ni(NO_3)_2$ for 24 h. The SD values are shown.

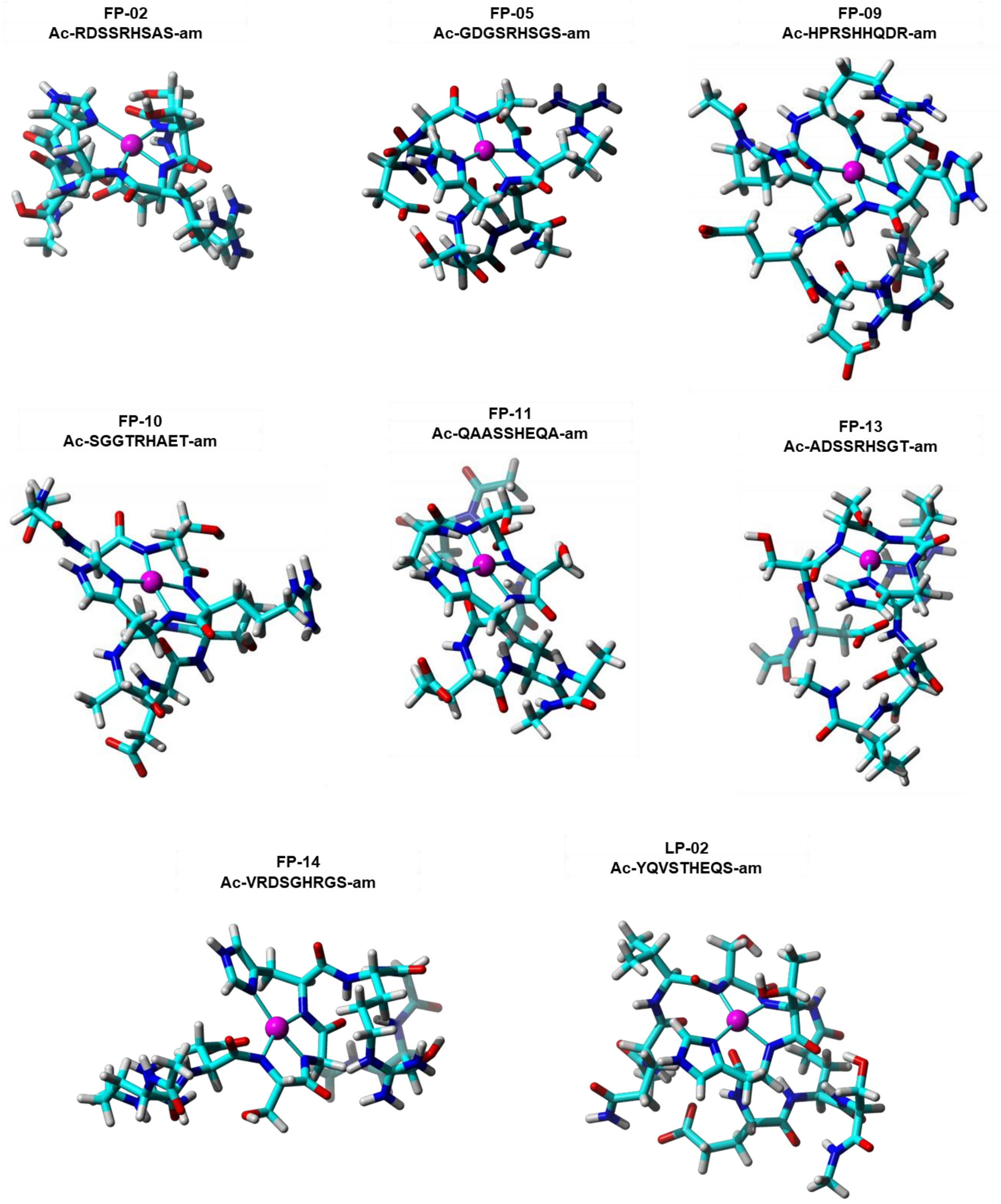

Supplementary Figure 2. Molecular modelling of filaggrin peptides (FPs). Atoms are marked in colours as: cyan (C), red (O), white (H), indigo (N) and magenta (Ni).

FP-02
Ac-RDSSRHSAS-am

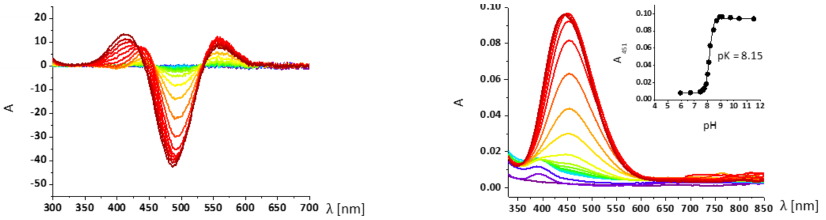

FP-05
Ac-GDGSRHSGS-am

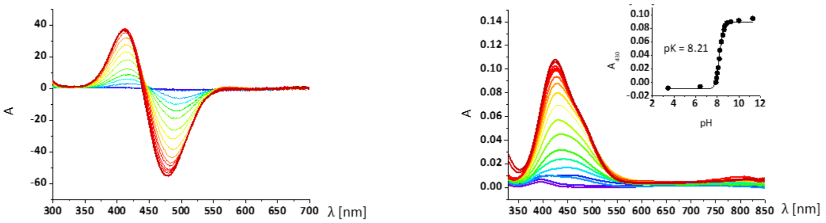

FP-09
Ac-HPRSHHQDR-am

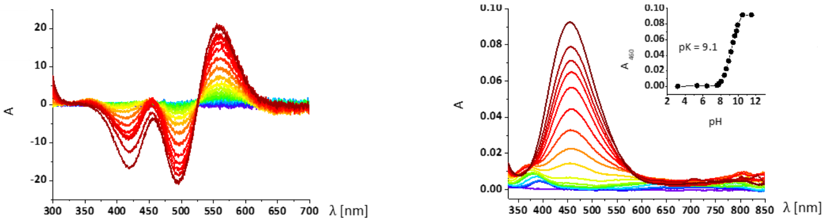

FP-10
Ac-SGGTRHAET-am

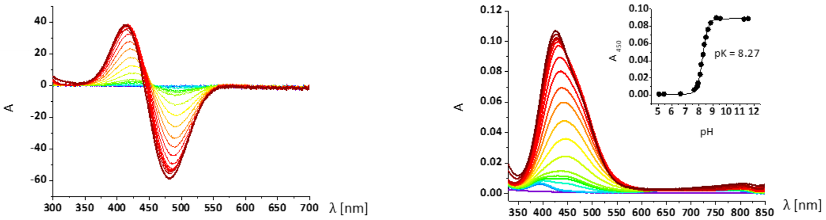

FP-11
Ac-QAASSHEQA-am

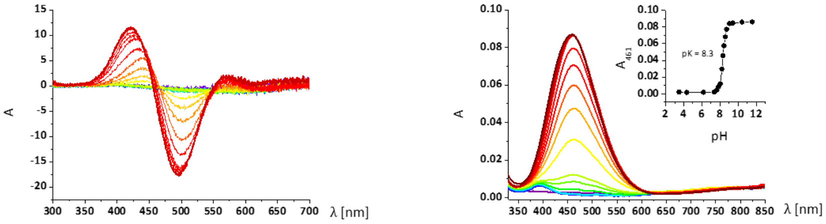

FP-13
Ac-ADSSRHSGT-am

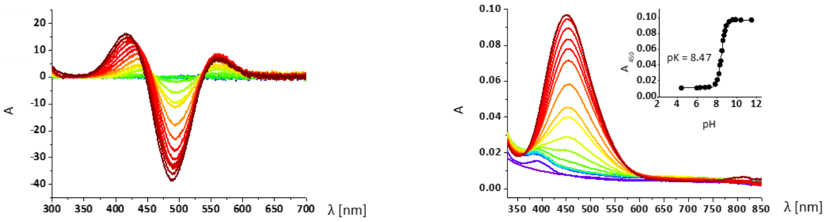

FP-14
Ac-VRDSGHRGS-am

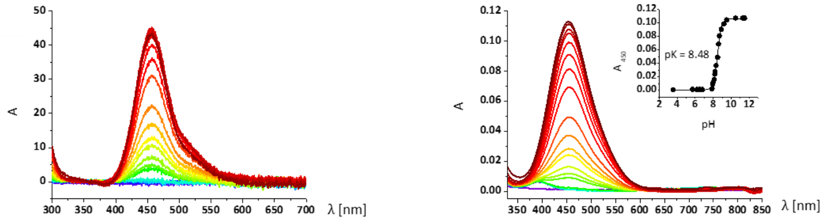

FLP-02
Ac-YQVSTHEQS-am

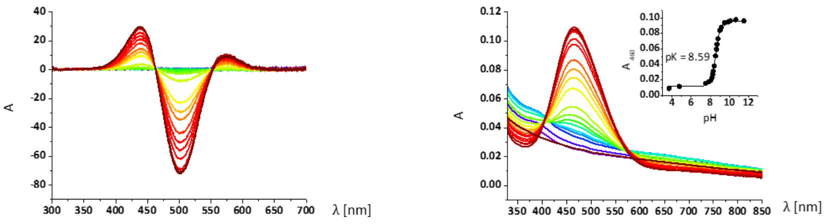

Supplementary Figure 3. CD pH titration and UV-VIS pH titration curves for nickel-peptide complexes. The pH values marked with colour gradient from dark blue (the lowest pH=3.5) to red (the highest pH=11.5).

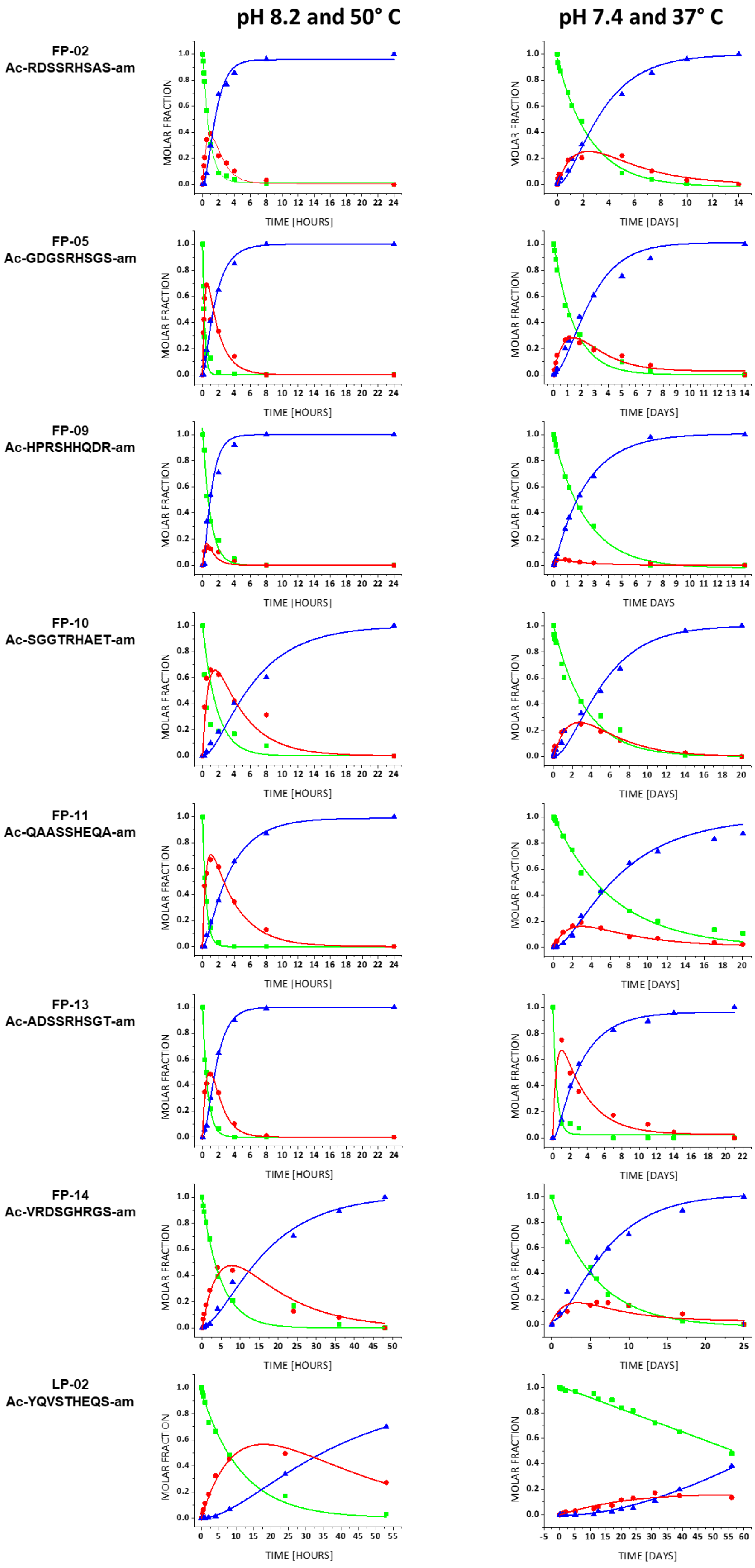
pH 8.2 and 50° C
pH 7.4 and 37° C
FP-02
Ac-RDSSRHSAS-am
FP-05
Ac-GDGSRHSGS-am
FP-09
Ac-HPRSHHQDR-am
FP-10
Ac-SGGTRHAET-am
FP-11
Ac-QAASSHEQA-am
FP-13
Ac-ADSSRHSGT-am
FP-14
Ac-VRDSGHRGS-am
LP-02
Ac-YQVSTHEQS-am
MOLAR FRACTION
TIME [HOURS]
TIME [DAYS]
TIME DAYS

Supplementary Figure 4. Kinetic studies for hydrolysis in the pH 8.2, 50° C and pH 7.4 and 37° C; green squares (substrate), red circles (intermediate product), indigo triangles (final products).

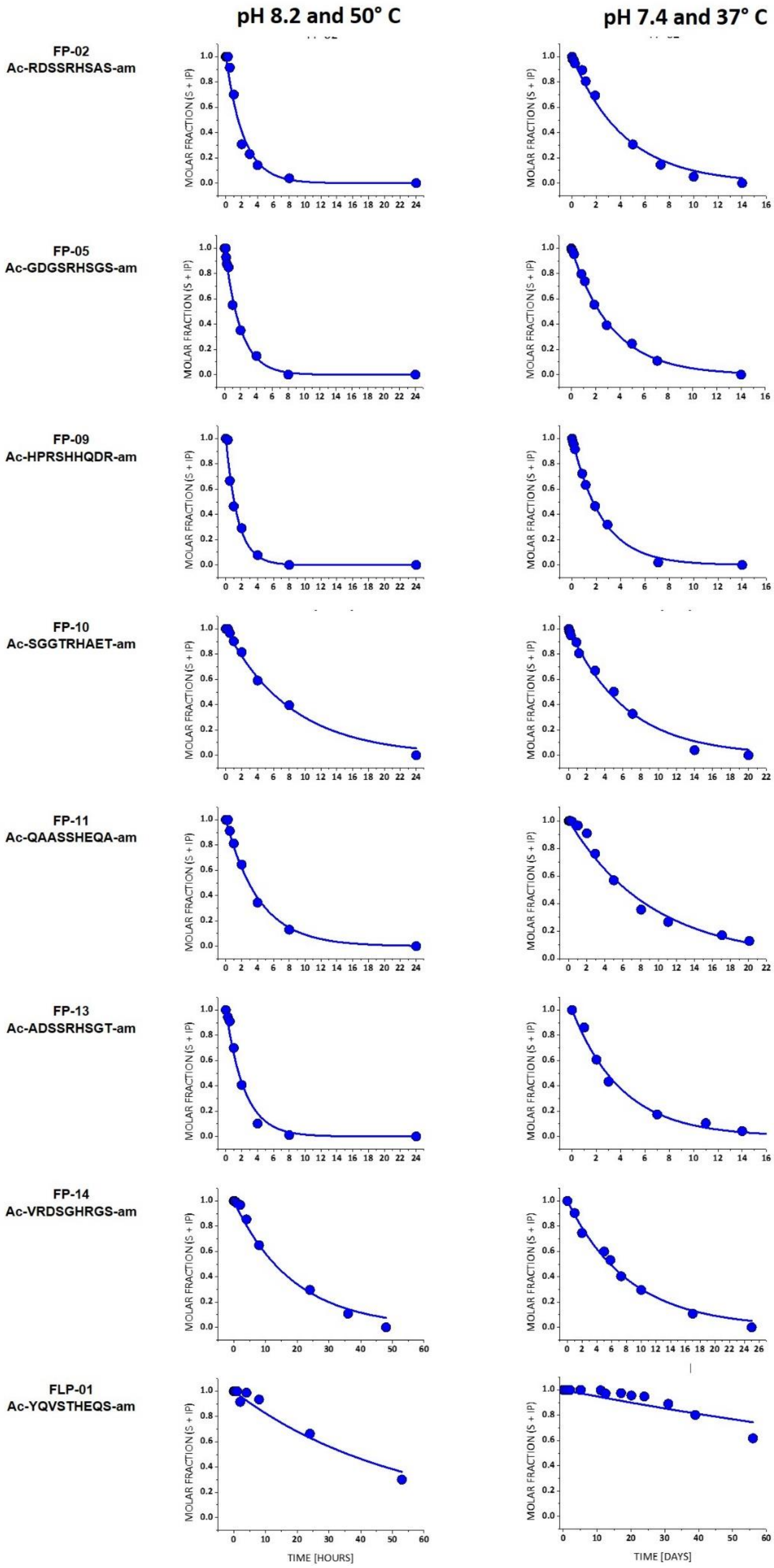
pH 8.2 and 50° C
pH 7.4 and 37° C
FP-02
Ac-RDSSRHSAS-am
FP-05
Ac-GDGSRHSGS-am
FP-09
Ac-HPRSHHQDR-am
FP-10
Ac-SGGTRHAET-am
FP-11
Ac-QAASSHEQA-am
FP-13
Ac-ADSSRHSGT-am
FP-14
Ac-VRDSGHRGS-am
FLP-01
Ac-YQVSTHEQS-am
MOLAR FRACTION (S + IP)
TIME [HOURS]
TIME [DAYS]

Supplementary Figure 5. Curve fitting results for the sum of the substrates (S) and intermediate products (IP) upon filaggrin peptide (FP) hydrolysis under harsh and physiological conditions (pH 7.4 and 37 °C).

A
B
C
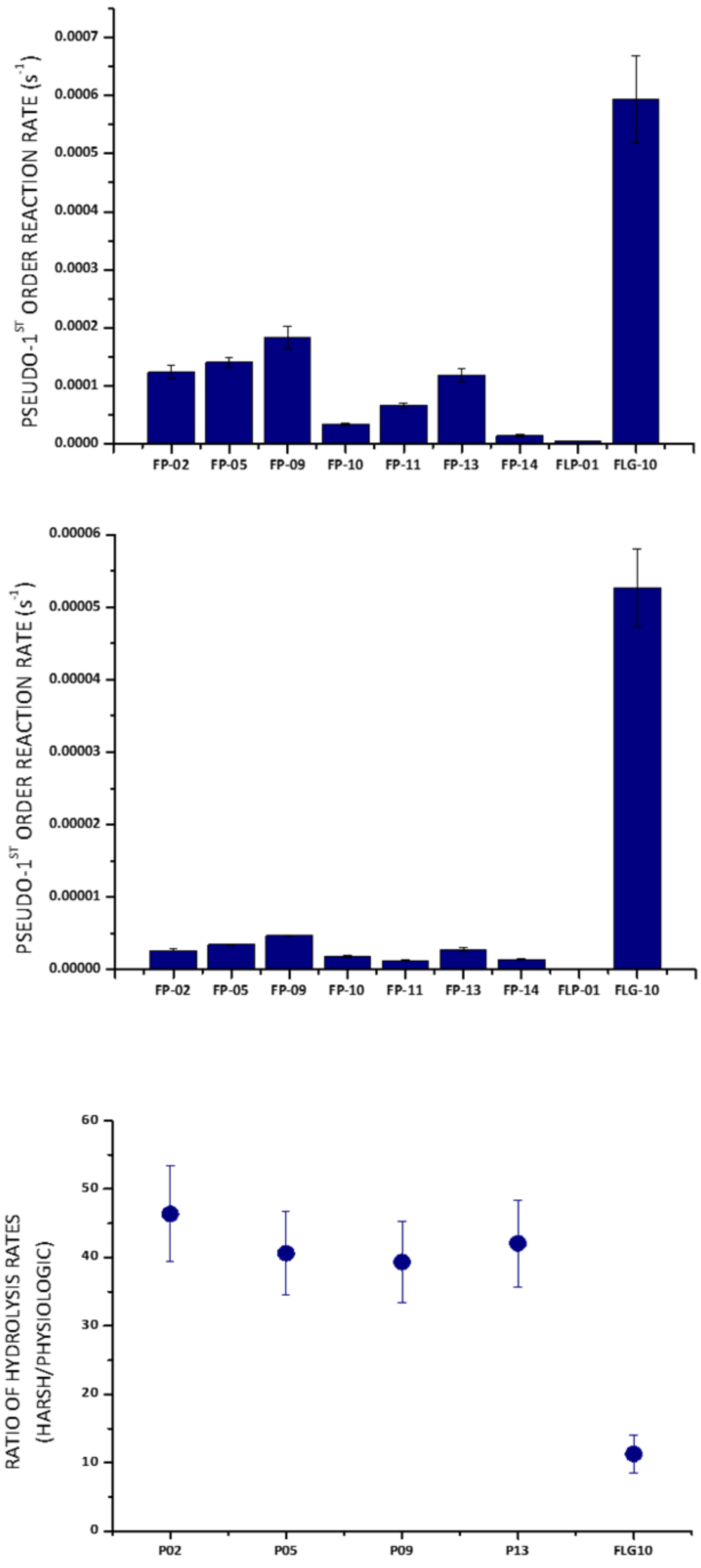
PSEUDO-1ST ORDER REACTION RATE (s-1)
0.0000
0.0001
0.0002
0.0003
0.0004
0.0005
0.0006
0.0007
FP-02
FP-05
FP-09
FP-10
FP-11
FP-13
FP-14
FLP-01
FLG-10
PSEUDO-1ST ORDER REACTION RATE (s-1)
0.00000
0.00001
0.00002
0.00003
0.00004
0.00005
0.00006
FP-02
FP-05
FP-09
FP-10
FP-11
FP-13
FP-14
FLP-01
FLG-10
RATIO OF HYDROLYSIS RATES
(HARSH/PHYSIOLOGIC)
0
10
20
30
40
50
60
P02
P05
P09
P13
FLG10

Supplementary Figure 6. (A) A comparison of Ni^{2+}-assisted hydrolysis rates for filaggrin peptides (FP) and recombinant filaggrin monomer (FLG-10) obtained for harsh conditions (pH 8.2 and 50 °C). **(B)** A comparison of Ni^{2+}-assisted hydrolysis rates for FPs and FLG-10) obtained for physiological conditions (pH 7.4 and 37 °C). **(C)** The comparison of reaction rate decrease upon switch from harsh to physiological conditions for the most active model peptides and the FLG-10 domain. The difference between the peptides and the protein domain indicates higher susceptibility to hydrolysis.

HP-02
SHHEQARDS

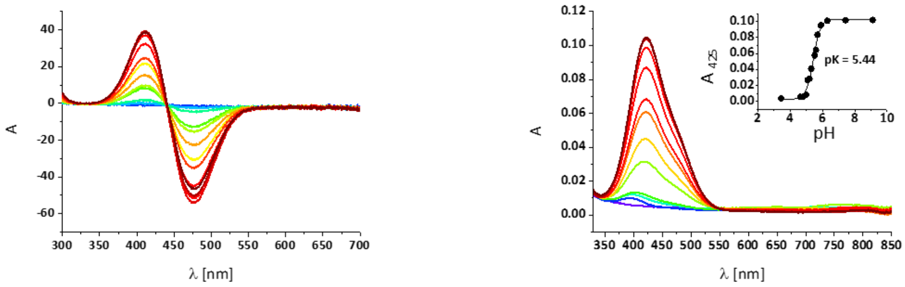

HP-06
SRHSG

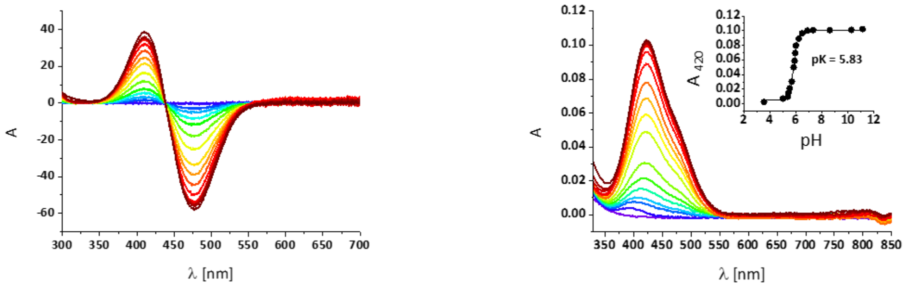

HP-07
SRHHEASSADS

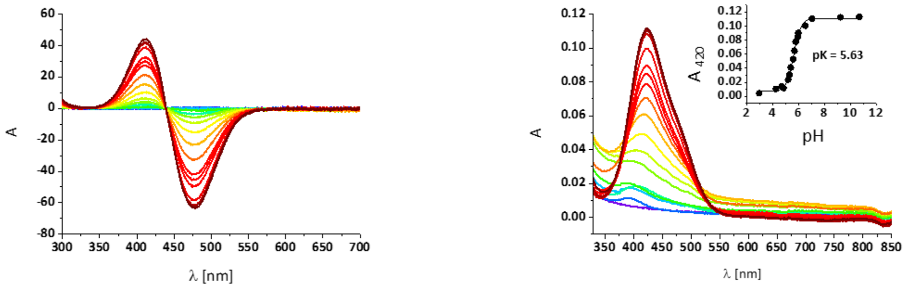

HP-12
SRHSGIGHGQASSAVRD

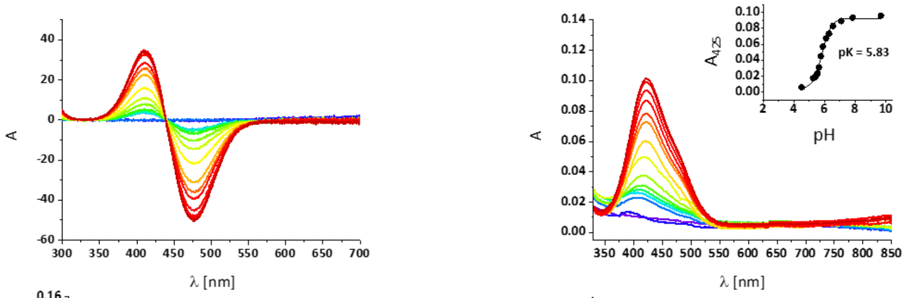

HP-13
SRHSGIGHGQASSAVRD

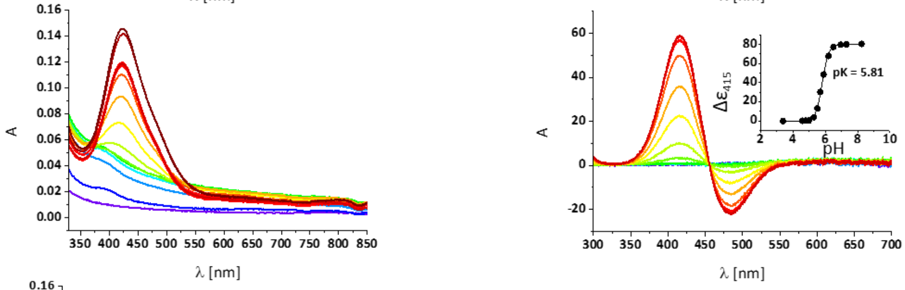

HP-14
SAHGQAGSHQQSHQESARGRSGET

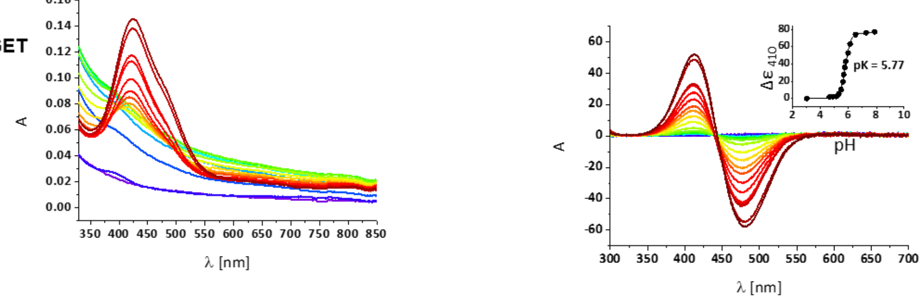

Supplementary Figure 7. CD and UV-VIS pH titration for the FLG hydrolysis products (HPs) complexed with nickel. The pH values marked with color gradient from dark blue (the lowest pH=3.5) to red (the highest pH=11.5).

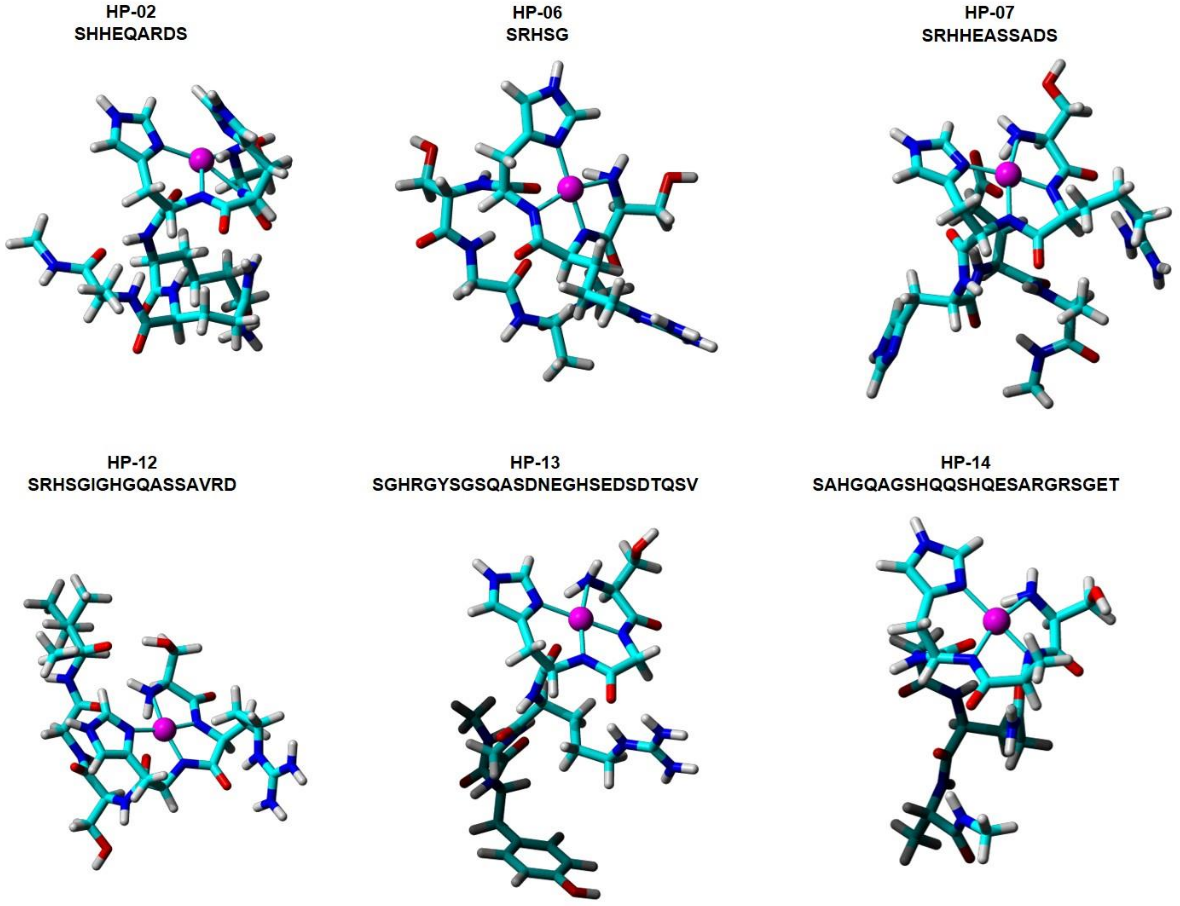

Supplementary Figure 8. Molecular modelling of the FLG hydrolysis products (HPs) complexed with nickel. Atoms are marked in colours as: cyan (C), red (O), white (H), indigo (N) and magenta (Ni).

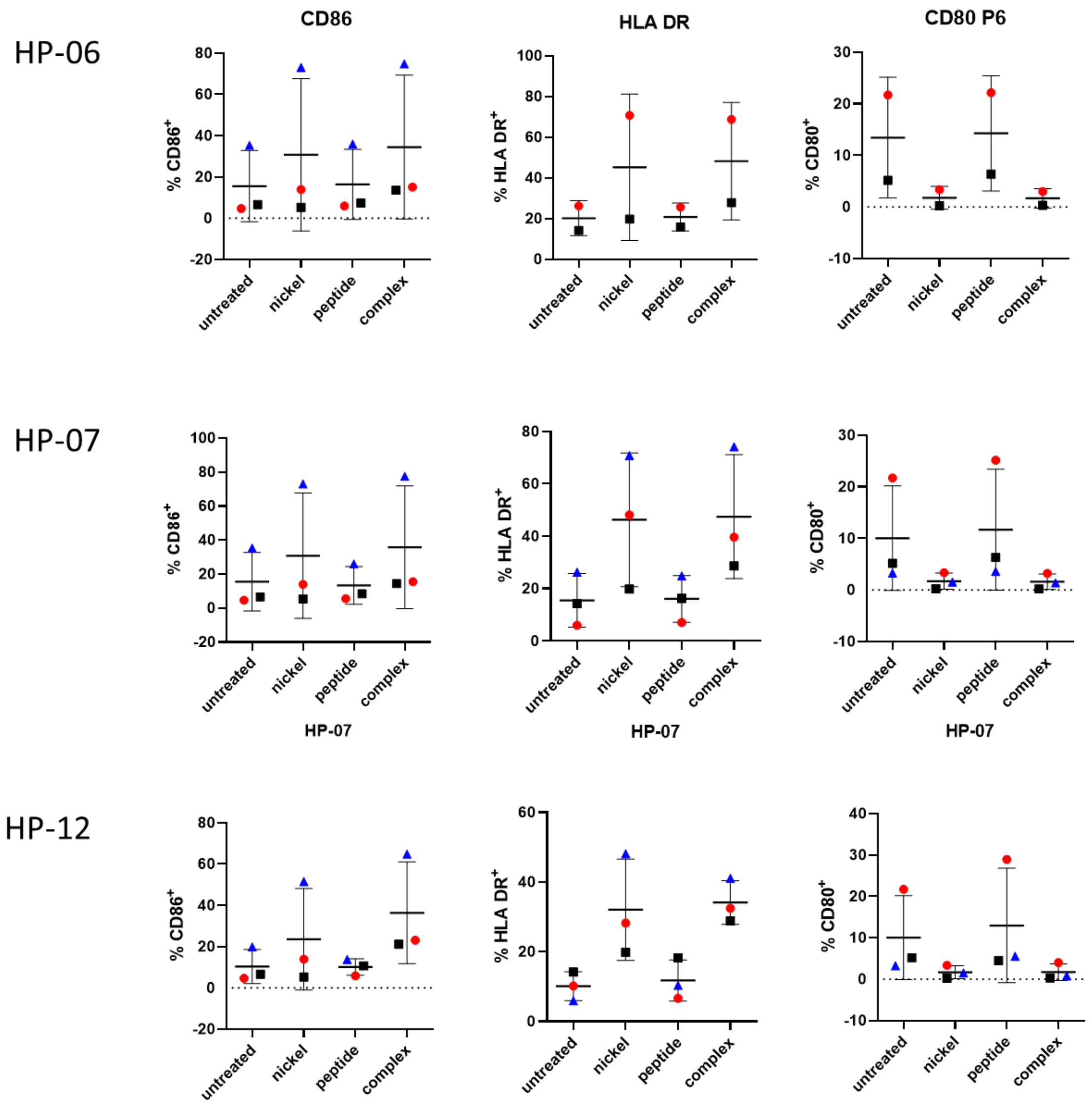

Supplementary Figure 9. MDLCs response to Ni-HPs, the proposed products of Ni-driven FLG hydrolysis. Changes in phenotype after exposure to$NiSO_4$, HP-06, HP-07, HP-12, FLG-10 domain and their Ni^{2+} complexes for 48 hrs are shown. Data from three separate biological experiments (n=3) ; means and SD values are shown.

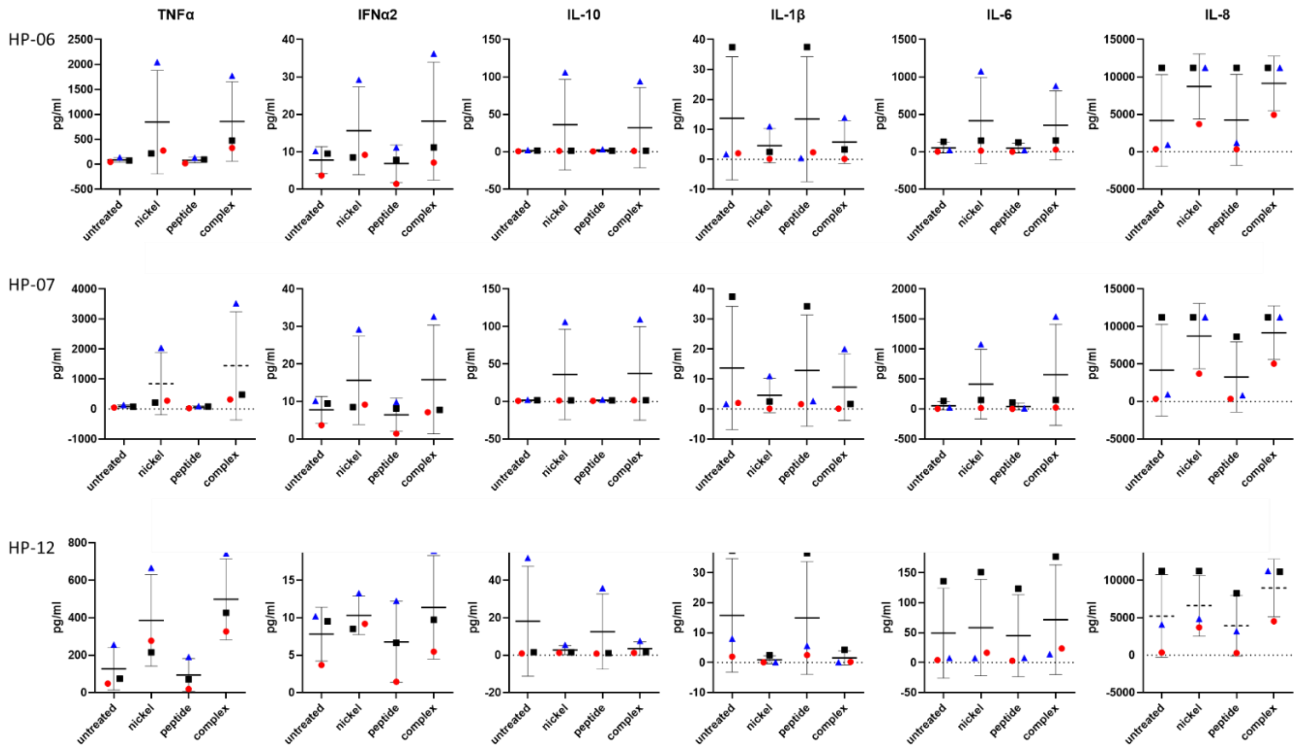

Supplementary Figure 10. MDLCs response to Ni-HPs, the proposed products of Ni-driven FLG hydrolysis. Changes in secreted cytokine levels after exposure to$NiSO_4$, HP-06, HP-07, HP-12, and their Ni^{2+} complexes for 48 hrs are shown. Data from three separate biological experiments (n=3) ; means and SD values are shown.

3 Supplementary References

Bal, W., Kozlowski, H., Robbins, R., and Pettit, L. D. (1995). Competition between the terminal amino and imidazole nitrogen donors for coordination to Ni(II) ions in oligopeptides. Inorganica Chimica Acta 231, 7–12. doi:10.1016/0020-1693(94)04333-q.

Bal, W., Lukszo, J., Bialkowski, K., and Kasprzak, K. S. (1998). Interactions of Nickel(II) with histones: interactions of Nickel(II) with CH3CO-Thr-Glu-Ser-His-His-Lys-NH2, a peptide modeling the potential metal binding site in the “C-Tail” region of histone H2A. Chem. Res. Toxicol. 11, 1014–1023.

Kopera, E., Krężel, A., Protas, A. M., Belczyk, A., Bonna, A., Wysłouch-Cieszyńska, A., et al. (2010). Sequence-Specific Ni(II)-Dependent Peptide Bond Hydrolysis for Protein Engineering: Reaction Conditions and Molecular Mechanism. Inorganic Chemistry 49, 6636–6645. doi:10.1021/ic1005709.

Kozłowski, H., Bal, W., Dyba, M., and Kowalik-Jankowska, T. (1999). Specific structure–stability relations in metallopeptides. Coordination Chemistry Reviews 184, 319–346. doi:10.1016/s0010-8545(98)00261-6.

Mylonas, M., Krężel, A., Plakatouras, J. C., Hadjiliadis, N., and Bal, W. (2002). The binding of Ni(ii) ions to terminally blocked hexapeptides derived from the metal binding -ESHH- motif of histone H2A. J. Chem. Soc., Dalton Trans., 4296–4306. doi:10.1039/b206585a.

Sunderman, F. W., William Sunderman, F., Varghese, A. H., Kroftova, O. S., Grbacivankovic, S., Kotyza, J., et al. (1996). Characterization of pNiXa, a serpin ofXenopus laevis oocytes and embryos, and its histidine-rich, Ni(II)-binding domain. Molecular Reproduction and Development 44, 507–524. doi:10.1002/(sici)1098-2795(199608)44:4<507::aid-mrd11>3.0.co;2-v.

Wilkins, M. R., Gasteiger, E., Bairoch, A., Sanchez, J. C., Williams, K. L., Appel, R. D., et al. (1999). Protein identification and analysis tools in the ExPASy server. Methods Mol. Biol. 112, 531–552.
